# FOXI3 establishes the ectodermal niche in pharyngeal arches for cranial neural crest cells and their lineages

**DOI:** 10.1038/s41413-025-00499-w

**Published:** 2026-02-04

**Authors:** Xin Chen, Siyi Wu, Ying Chen, Chenlong Li, Xingmei Feng, Yaoyao Fu, Yongchang Zhu, Yiyuan Chen, Lin Chen, Run Yang, Ranran Dai, Jing Zhang, Aijuan He, Xin Wang, Duan Ma, Bingtao Hao, Tianyu Zhang, Jing Ma

**Affiliations:** 1 ENT Institute, Department of Facial Plastic and Reconstructive Surgery, Eye & ENT Hospital, Fudan University, Shanghai, China; 2 Affiliated Hospital of Nantong University, Nantong, China; 3 Department of Immunology, School of Basic Medical, Zhengzhou University, Zhengzhou, Henan China; 4 Zhongshan School of Medicine, Sun Yat-Sen University, Guangzhou, Guangdong China; 5 Key Laboratory of Metabolism and Molecular Medicine, Ministry of Education, Department of Biochemistry and Molecular Biology, School of Basic Medical Sciences, Fudan University, Shanghai, China; 6 Henan Eye Institute, Henan Academy of Innovations in Medical Science, Zhengzhou, Henan China; 7 NHC Key Laboratory of Hearing Medicine (Fudan University), Shanghai, China; 8 Institute of Medical Genetics & Genomics, Fudan University, Shanghai, China; 9 Shanghai Key Laboratory of Gene Editing and Cell Therapy for Rare Diseases, Shanghai, China

**Keywords:** Pathogenesis, Diseases

## Abstract

Craniofacial development relies on the migration of cranial neural crest cells (CNCCs) to the first and second pharyngeal arches, followed by their differentiation into various cell types during embryogenesis. Although the CNCC migration has been well-studied, the role of the niche in relation to CNCC remains unclear. Variants in *FOXI3* have been implicated in craniofacial microsomia (CFM), yet the molecular mechanisms remain unexplored. FOXI3 is expressed in the ectoderm and auricle epidermis, but not in CNCCs or cartilage. Deletion of *Foxi3* in the mouse CNCCs did not disrupt mandible and auricular development, further confirming that FOXI3 does not directly regulate CNCCs. However, *Foxi3* deficiency in the ectoderm reduced the production of chondrogenesis-related cytokines derived from ectodermal cells, such as TGF-β1. This impairment affected CNCC proliferation through cell communication, subsequently altering the development of the mandible and auricle. These results emphasize the critical role of FOXI3 in establishing the microenvironment supporting CNCC function. Furthermore, FOXI3 directly regulates target genes associated with translation, thereby orchestrating cytokine production in epidermal cells. The validation using auricle sample from a CFM patient carrying *FOXI3* mutation further supports our findings. These insights highlight the function of FOXI3 in creating the niche necessary for CNCC development and provide a basis for understanding the molecular mechanisms driving CFM pathogenesis.

## Introduction

The pharyngeal arch represents a transient embryonic structure, consisting of an outer ectoderm, an inner endoderm, and a central mesoderm. Cranial neural crest cells (CNCCs) originate from the border between the neural plate and surface ectoderm, delaminate from the dorsal epithelium, and migrate to the mesodermal mesenchyme of the first and second pharyngeal arches, subsequently differentiating into various craniofacial skeletal cell types, including chondrocytes and osteoblasts.^1^ The pharyngeal arches serve as a specialized microenvironment, or niche, for CNCCs, significantly influencing their proliferation and differentiation.^2^ Within this niche, cytokines such as Wnt, BMP, FGF, and TGF-beta play necessary roles in CNCC development.^3^ Although the critical functions of signaling molecules and transcription factors in ectodermal or endodermal cells of the pharyngeal arches for arch patterning have been recognized,^4^ the precise molecular mechanisms involved remain incompletely understood.

FOXI3, a member of the forkhead box transcription factor family,^5^ emerges as a key regulator of embryonic development, influencing the formation of organs and tissues, including the ear,^6,7^ thymus,^8^ and skin appendages.^9^ Previous studies have highlighted its importance in ectodermal development, demonstrated by Peruvian hairless dogs with a spontaneous *FOXI3* mutation, which exhibit sparse fur coats and missing teeth.^9,10^ These dogs also present mesodermal structural anomalies, including auricle malformation and external auditory canal stenosis.^11^ Similarly, *Foxi3*-deficient mice show complete absence of the mandible, as well as external and middle ear structures,^12^ further underscoring the critical roles of FOXI3 in mesoderm development.

Craniofacial microsomia (CFM), also known as Goldenhar syndrome, Oculo-Auriculo-Vertebral spectrum, or hemifacial microsomia, is a craniofacial morphogenetic disorder with an incidence ranging from 1 in 5 600 to 1 in 3 500 live births.^6^ This condition typically manifests as microtia, preauricular tags and pits, and facial asymmetry, all associated with abnormal development of the first and second pharyngeal arches and CNCCs.^13^ Microtia, the most common and mildest form of CFM,^14^ typically presents with auricle deformities, often accompanied by malformations of the external auditory canal and middle ear.^15^

In humans, a microdeletion encompassing the *FOXI3* gene has been reported in an individual with microtia and ipsilateral internal carotid artery agenesis.^11^ Additionally, recurrent deletions at 2p11.2, which overlap the *FOXI3* gene, have been identified in five pedigrees with DiGeorge syndrome features, including ear malformations.^8^ Recent investigations have also implicated pathogenic variants in *FOXI3* in CFM pedigrees.^6,7^ However, despite these associations, the molecular mechanisms by which FOXI3 contributes to CFM pathogenesis remain unclear. While the cartilage of the external and middle ear, the ossicles of the middle ear, and the mandible originate from CNCCs in the first and second pharyngeal arches,^16^ FOXI3 expression is conspicuously absent in CNCCs,^12,17,18^ suggesting that FOXI3 may exert a cell non-autonomous effect on CNCC survival.^12^

As a transcription factor, FOXI3 has been shown to activate the expression of the target gene *AE4* of FOXI1 by binding to its promoter when artificially expressed in HEK293 cells.^19^ Notably, FOXI3 has also been identified as a 5-formylcytosine-binding protein, suggesting a role in transcription and chromatin regulation.^20^ Additionally, FOXI3 has been proposed as a novel SMAD cofactor, required for SMAD-dependent transcriptional regulation in the TGF-beta signaling pathway.^21^ Therefore, despite evidence supporting FOXI3’s key regulatory role in the development of the external and middle ear, as well as the mandible, critical questions linger regarding the specific cell types harnessing FOXI3 to orchestrate cartilage development and the target genes subject to FOXI3’s regulation in these processes.

In this study, the expression pattern of FOXI3 and its molecular role in CFM were investigated. It was demonstrated that FOXI3 is specifically expressed in the ectoderm of the first and second pharyngeal arches and auricle epidermis. Deletion of *Foxi3* in CNCCs and their lineage cells did not affect mouse mandible and auricular development. However, *Foxi3* deficiency in the ectoderm and ectoderm-derived epidermal cells disrupted cytokine-mediated cell communication between the ectoderm and CNCCs, impairing the microenvironment of CNCC development, inhibiting CNCC proliferation, and ultimately leading to auricular underdevelopment and mandibular hypoplasia. Interestingly, FOXI3 within the ectoderm and ectoderm-derived epidermis does not directly regulate chondrogenesis-related cytokines transcription but controls their translation. These findings are further validated through analysis of a CFM patient sample carrying a heterozygous *FOXI3* frameshift mutation. Collectively, we underscore the importance of FOXI3 in the ectodermal niche in the first and second pharyngeal arches for CNCC development and enhance our understanding of FOXI3’s biological role in both normal and pathological developmental contexts, addressing critical gaps in the field.

## Results

### *Foxi3* deficiency in mouse cranial neural crest cells does not affect the development of the mandible, external, and middle ear

**Fig. 1:**
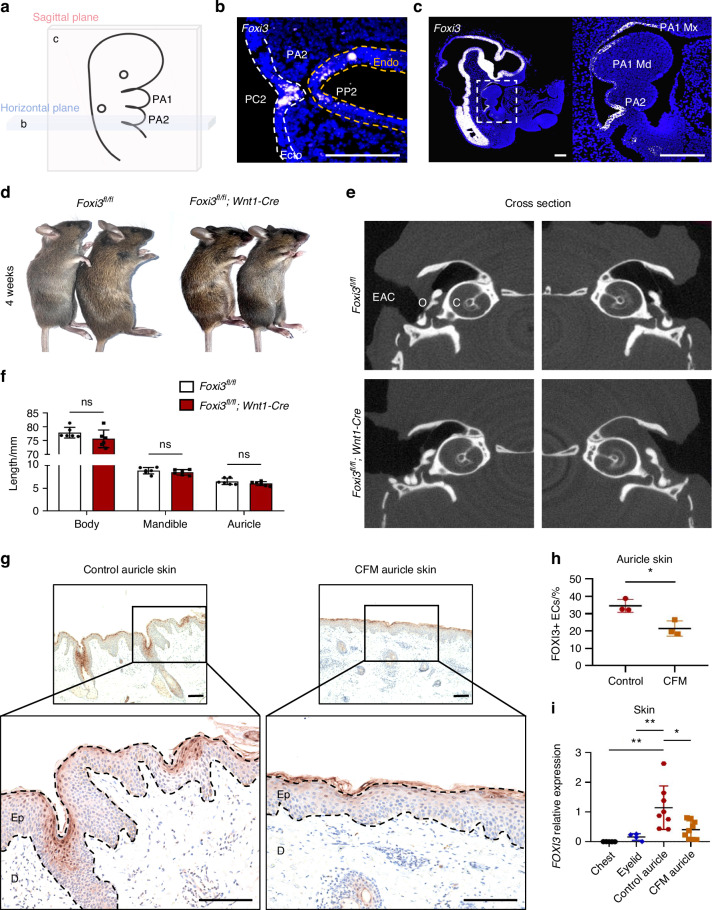
FOXI3 is specifically expressed in the ectoderm and auricle skin, and its deficiency in mouse CNCCs does not affect craniofacial development. **a** Schematic of anatomical planes in E10.5 mouse embryo. **b**, **c** RNAscope in situ hybridization revealing *Foxi3* mRNA expression. Transverse section (**b**) demonstrates *Foxi3* expression within the ectoderm (Ecto, white dashed lines) and endoderm (Endo, yellow dashed lines) of the second pharyngeal arch (PA2), particularly in the second pharyngeal cleft (PC2) and pouch (PP2). Longitudinal section (**c**) and magnified view reveal *Foxi3* expression in the neural tube neuroepithelium and the ectoderm of the PA1 (maxilla, Mx; mandible, Md) and PA2, but not in the mesodermal mesenchyme filled with CNCCs. Scale bars: 200 µm. **d** CNCC-specific *Foxi3* conditional knockout (cKO) and control littermates exhibit normal mandibles and auricles at 4 weeks. **e** Quantification of body, mandible, and auricle lengths (*n* = 6/genotype). **f** Micro-CT shows normal external auditory canal (EAC), ossicles (O), and cochlea (C) in both groups. Immunohistochemistry (**g**) and quantification (**h**) of auricle skin reveal reduced FOXI3 expression in CFM patients, particularly in the epidermis (Ep). Scale bars: 250 µm (low-power), 50 µm (high-power). **i** qPCR detection of *FOXI3* expression in control skin (auricle, *n* = 8; eyelid, *n* = 4; chest, *n* = 5) and CFM patient auricle skin samples (*n* = 9). Statistical significance was analyzed via unpaired two-tailed Student’s *t* test for (**f**, **h**), one-way ANOVA with Tukey’s multiple comparison test for (**i**). ns no significance; *P < 0.05, **P < 0.01

Ohyama et al. and Edlund et al. employed whole-mount in situ hybridization to map *Foxi3* transcripts during early mouse embryogenesis. Their findings reveal that prior to and during pharyngeal arch development, *Foxi3* is widely expressed in both the ectoderm and endoderm, while no *Foxi3* signal was detected in the mesoderm or in the CNCC-derived mesenchyme.^12,17^ In our study, we obtained consistent results through RNAscope in situ hybridization analysis of pharyngeal arch tissue sections from E10.5 mouse embryos. *Foxi3* is expressed in the neuroepithelium of the neural tube, as well as in the ectoderm of the first and second pharyngeal arches, but not in the mesodermal mesenchyme filled with CNCCs (Fig. 1a–c). A recent study investigated the expression pattern of *Foxi3* during early embryonic development using two mouse models: *Foxi3*^*GFP*^ mice, designed to detect *Foxi3* expression across various developmental stages, and *Foxi3*^*CreER*^ mice, used for tracing *Foxi3*-labeled cell derivatives.^18^ The study demonstrated that FOXI3 is prominently present in the ectoderm of the first and second pharyngeal arches, with its derivatives observed in the epidermis of the auricle and external auditory canal.^18^ Importantly, FOXI3 expression was absent in the CNCCs that contribute to the mesodermal mesenchyme of the first and second pharyngeal arches, as well as in the cartilage of the auricle and external auditory canal.^12,17,18^

*Foxi3*-null mice exhibited severe facial skeletal defects, including the absence of the mandible and ear structures.^12^ To determine whether transient FOXI3 expression in CNCCs influenced development, CNCC-specific *Foxi3* conditional knockout (cKO) mice (*Foxi3*^fl/fl^; *Wnt1*-Cre) were generated (Fig. S1a, b). Remarkably, homozygous *Foxi3* cKO mice developed without visible abnormalities in the mandible, auricles (Fig. 1d, e), external auditory canals, ossicles, or cochlea (Fig. 1f).

Further investigation of various cell lines revealed minimal *FOXI3* expression in human mesenchymal stem cells (MSCs) and cartilage cells (Fig. S2a). Conversely, significant *FOXI3* expression was detected in human embryonic stem cells (ESCs), implying a potential role during early embryogenesis. Additionally, *FOXI3* was found to be expressed in two epidermal cell lines, namely the human epidermal squamous-cell carcinoma cell line A431 and the human keratinocyte line HaCaT (Fig. S2a).

Given the common occurrence of auricle malformation in CFM, human auricle skin samples were collected for analysis (Fig. 1g–i). FOXI3 was primarily localized in the epidermal layer, as revealed by morphological assessments (Fig. 1g). Strikingly, *FOXI3* showed high expression levels in auricle skin but was hardly detectable in other skin tissues (Fig. 1i). Consistent with findings by Khatri SB et al., which reported that *Foxi3* expression in mice was restricted to the epithelium during ectodermal appendage development and undetectable in trunk epidermis,^22^ we confirmed that *FOXI3* expression was significantly diminished in the chest skin of human samples. Notably, in patients with CFM, FOXI3 expression in auricle skin, especially within the epidermis, was markedly lower compared to controls (Fig. 1g–i).

These findings suggest that the pathogenic mechanism of *FOXI3* in CFM differs from those of known CFM-causing genes such as *SF3B2*,^23^ *HOXA1*,^24^ *HOXA2*,^25^ and *TBX1*,^26^ which are expressed in CNCCs and directly regulate CNCC development.

### Auricle skin cells communicate with chondrocytes through chondrogenesis-related cytokines

FOXI3’s distinct expression pattern and the proximity between the ectoderm and CNCC in the pharyngeal arch, as well as between the skin and cartilage in the auricle,^27,28^ suggest a role for specific FOXI3-expressing cells in coordinating auricle development through intercellular communication. Neighboring cells typically use cytokines as mediators for communication,^29^ with several cytokines, including TGF-βs, FGFs, and WNTs, being key in CNCC development and chondrogenesis.^3^ This highlights the necessity of balanced cytokine signaling to ensure proper cartilage formation.

The auricle undergoes continuous growth from the embryonic period through the postnatal stage. To investigate skin-chondrocyte interactions and identify relevant cytokines, single-cell RNA sequencing (scRNA-seq) was conducted on human auricle skin and cartilage samples from one 13-week gestation fetus (a stage marking the morphological transition of the outer ear from the upper neck to the lateral head) and three teenagers (representing the postnatal stage when the auricle achieves mature morphology) without developmental abnormalities. Following stringent cell filtration, 14 477 auricle skin cells and 28 970 auricle cartilage cells were retained for co-analysis (Fig. S3a). Using uniform manifold approximation and projection (UMAP), based on specific marker expression, six cell subtypes were identified: chondrocytes, basal cells (BC), spinous cells (SC), fibroblasts (FB), pericytes (PC), and endothelial cells (EC) (Fig. S3a–c). Marker gene expression for each subtype aligned with classical markers (Fig. S3b, c). Gene Ontology (GO) analysis of biological processes (BP) for each cell subpopulation was consistent with known functions (Fig. S3d). The proportions of chondrocytes and major skin cell subtypes (FB, BC, and SC) were similar across the three teenage samples (Fig. S3e).

**Fig. 2:**
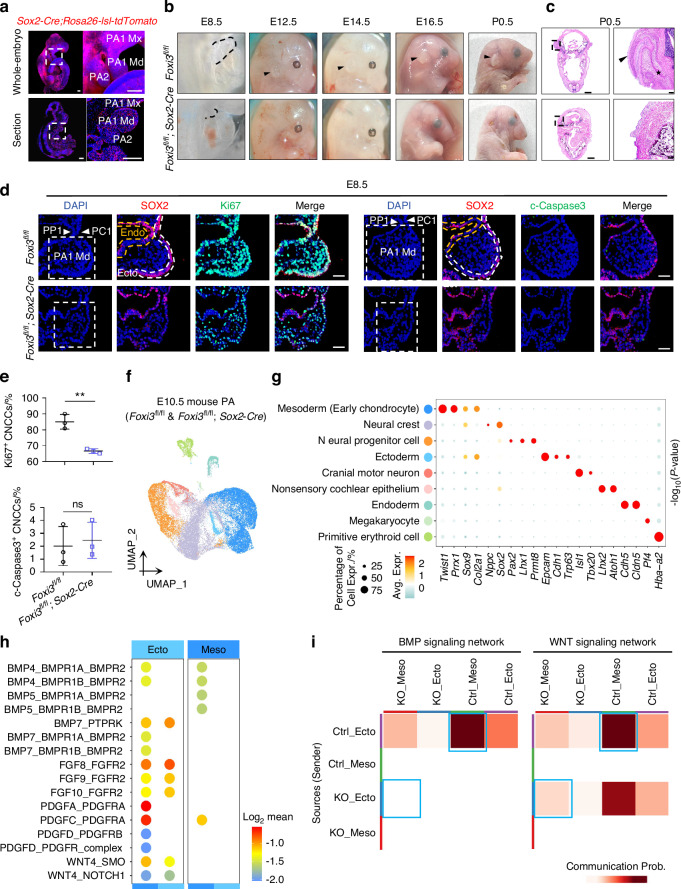
*Foxi3* deficiency in the ectoderm inhibits CNCC proliferation through cellular communication. **a** Whole-embryo clearing and 3D light-sheet imaging of an E10.5 *Sox2-Cre*; *Rosa26-lsl-tdTomato* embryo showing widespread recombination, including the pharyngeal arch (PA) region (dashed box). The longitudinal section and magnified view reveal tdTomato-expressing cells in both ectoderm and mesoderm of the PA1 (maxilla, Mx; mandible, Md) and PA2. Scale bars: 200 µm. **b** Gross inspections of *Foxi3*^fl/fl^; *Sox2-Cre* mice demonstrate smaller PA (E8.5), auricle absence, and mandibular hypoplasia (E12.5-P0.5), with consistent magnification across samples. **c** H&E staining of *Foxi3*^fl/fl^; *Sox2-Cre* mice reveals the absence of external ears at P0.5. Arrows mark auricles; asterisks indicate external auditory canals. Scale bars: 1 000 µm (low magnification) and 100 µm (high magnification). **d** Immunofluorescence of E8.5 *Foxi3*^fl/fl^; *Sox2-Cre* mouse sections reveals smaller PAs, structural disorganization in the ectoderm (Ecto), and a significant reduction in PA cell proliferation. **e** Quantification indicates a significant decrease in SOX2^-^ CNCCs, with no change in apoptosis. Scale bars, 50 µm. **f** UMAP of single-cell data from E10.5 *Foxi3*^fl/fl^; *Sox2-Cre* and control PAs identifies distinct cell populations. **g** Dot plot shows expression of cluster-specific marker genes; dot size denotes expression fraction, and color intensity indicates mean scaled expression. **h** Dot plot of chondrogenesis-related ligand-receptor pairs in ectoderm and mesoderm cells of *Foxi3*^fl/fl^; *Sox2-Cre* and control embryos, with color indicating interaction scores. **i** CellPhoneDB analysis illustrates weakened BMP/WNT signaling between ectoderm and mesoderm in *Foxi3*-deficient (KO) embryos (blue boxes). The vertical axis represents the “sender” population; darker colors indicate stronger communication probability. ns no significance; **P < 0.01 by unpaired two-tailed Student’s *t* test

In the embryonic stage, FBs dominated, accounting for approximately 48.2% of the total cell population (Fig. 2e). By adolescence, FBs prevalence declined to 11.9%, while BCs increased to approximately 4.6% (Fig. S3e). To gain insights into skin-cartilage communication, ligand-receptor interactions were analyzed using CellPhoneDB (Fig. S3f). The results indicated limited signaling from chondrocytes to skin cells, whereas skin cells produced an array of cytokines targeting chondrocytes. Although the cytokines present in the auricles of human fetus and teenagers were not identical, both groups include TGF-β1 and FGF7,^30–32^ which are closely associated with cartilage development. Notably, a greater variety of chondrogenesis-related cytokines were found in the fetus sample (Fig. S3f).

To further explore parallels in auricle development, scRNA-seq was performed on auricle tissues from E15.5 mice (corresponding to human 13-week embryos) and P7 mice (matching the human adolescent stage). After stringent cell filtration, 16 413 cells from E15.5 mice and 15 215 cells from P7 mice were included in the analysis (Fig. S4a). Cells were grouped according to marker genes and functions, and six distinct cell subtypes were visualized via UMAP (Fig. S4a–d). The proportion of BCs, FBs, and ECs exhibited significant changes (Fig. S4e). At E15.5, FBs accounted for approximately 85% of the cell population (Fig. S4e), while by P7, the proportion of FBs dropped to approximately 35%, and BCs increased to approximately 45% (Fig. S4e). the same subgroup in the auricle samples of E15.5 and P7 mice. This shift in FB and BC populations from embryonic to postnatal stages aligns with observations in humans (Fig. S3e). Ligand-receptor analysis demonstrated skin-cartilage communication through chondrogenesis-related cytokines also in both P7 and E15.5 mouse auricle (Fig. S4f). Additionally, an analysis of previously published mouse whole-embryo scRNA-seq data^33^ confirmed that the ectoderm secretes even higher quantities and a more diverse array of cytokines related to cartilage development, facilitating communication with CNCC-derived mesodermal cells during earlier stages of development (E9.0 to E13) (Fig. S4g).

The scRNA-seq findings from both mouse and human samples highlight the sustained communication between the ectoderm and CNCC-derived mesoderm during auricle development. These results indicate that auricle skin guides cartilage development through cytokines.

### *Foxi3* deficiency in mouse ectoderm and epidermis inhibits proliferation of CNCCs and their lineages by reducing the expression of ectoderm-derived chondrogenesis-related cytokines

This study identified robust cellular communication mediated by chondrogenesis-related cytokines between the auricle skin, originating from the ectoderm of the first and second pharyngeal arches, and the cartilage derived from CNCCs. Auricle morphogenesis in mice begins at E12, marked by the invagination of ectodermal cells into the mesodermal mesenchyme^16^ populated by CNCCs. In addition, the primary structure of the auricle is not derived from the endoderm.^34^ *Foxi3* is exclusively expressed in the ectoderm and endoderm, with no expression in the mesoderm or CNCCs, which contribute to the mesenchyme.^12^ The deficiency of *Foxi3* in CNCCs was shown not to impact mouse development (Fig. 1d–f), indicating that FOXI3’s effect on auricle development does not arise from directly influencing CNCCs. Consequently, this investigation focused on the niche provided by the ectodermal layer to support CNCC function.

*Sox2-Cre* has been reported to be expressed across all three germ layers during embryogenesis.^35^ Our analysis using whole-embryo tissue clearing, 3D light-sheet imaging, and histological sectioning of E10.5 *Sox2-Cre; Rosa26-lsl-tdTomato* double-transgenic mouse embryos revealed widespread recombination throughout the embryo. Specifically, tdTomato-expressing cells were detected in both the ectoderm and mesoderm of the pharyngeal arches (Fig. 2a).

To examine the effects of *Foxi3* deletion in the ectoderm on auricle and mandible development, *Foxi3*^fl/fl^; *Sox2*-Cre mice were generated. Immunofluorescence (IF) staining revealed that FOXI3 was almost completely knocked out in the auricle epidermis of E12.5 mice (Fig. S1a, c). Phenotypic observations were documented from E8.5 to P0.5 (Figs. 2b, c and S1c). At E8.5, smaller pharyngeal arches and structural disorganization in the ectoderm were exhibited by *Foxi3*^fl/fl^; *Sox2*-Cre (Fig. 2b, d). By E12.5, these mice exhibited underdeveloped auricles, characterized by a small protrusion where auricle was expected (Figs. 2b and S1c). Shortly after birth (P0.5), these mice were found to have died, demonstrating bilateral absence of auricles, underdeveloped external auditory canals, and mandibular hypoplasia (Fig. 2b, c). These observations were consistent with previous findings documented in *Foxi3* germline-deleted (*Foxi3*^−/−^) mice.^12^ Notably, significantly reduced CNCC proliferation was observed in *Foxi3*^fl/fl^; *Sox2*-Cre mice compared to control littermates, while no difference in apoptosis was detected (Fig. 2d, e).

Since cell communication persists during auricle development, co-culture experiments were performed using the epidermal cell line HaCaT (a permanent epithelial cell line derived from adult skin that can differentiate normally^36^ and secrete cytokines similar to those secreted by ectodermal cells) with MSCs or the chondrocyte line C28/I2 in vitro (Fig. S2b–d). HaCaT cells with knocked-down FOXI3 expression led to significantly reduced proliferation of MSCs and C28/I2 cells, while HaCaT cells overexpressing FOXI3 enhanced their growth (Fig. S2e, f). The auricle cartilage defect observed in patients with CFM is primarily related to decreased chondrocyte proliferation.^37^ No discernible effect of FOXI3 on apoptosis in MSCs or C28/I2 was observed in the co-culture system (Fig. S2g, h). These findings explain FOXI3’s role in promoting the proliferation of CNCCs and lineage cells through intercellular communication.

To confirm the existence of cellular communication between the ectoderm and mesoderm of the first and second pharyngeal arches, pharyngeal arch tissues were isolated from E10.5 *Foxi3*^fl/fl^; *Sox2*-Cre mice and their control littermates for scRNA-seq analysis (Fig. 2f). After the application of stringent cell filtration, a total of 28 242 cells were retained for subsequent analysis, comprising 12 933 cells from control littermates and 15 309 cells from *Foxi3*^fl/fl^; *Sox2*-Cre mice (Fig. 2f). Nine subpopulations were identified, including mesoderm (early chondrocytes), ectoderm, and endoderm clusters, based on specific marker expression. These clusters were visualized using UMAP (Fig. 2f, g). GO analysis of BP for each subpopulation was aligned with their respective functions (Fig. 2h). The mesoderm (early chondrocytes) cluster, characterized by the expression of established marker genes such as *Twist1*, *Prrx1*, *Sox9*, and *Col2a1*, corresponds to NCCs differentiating into cartilage (Fig. 2f, g). To gain insights into ectoderm-mesoderm communication, ligand-receptor interactions were analyzed using CellPhoneDB (Fig. 2h). Robust and unidirectional chondrogenesis-related signal transduction from ectodermal cells to mesodermal cells was indicated by the results, consistent with our findings from both human and mouse studies(Figs. S3f and S4f, g). The first publicly available single-cell atlas of the first and second pharyngeal arches in mice is also presented, providing a valuable resource for further exploration of craniofacial development. Moreover, *Foxi3* deficiency disrupts the cell crosstalk between ectoderm and mesoderm, particularly affecting the expression of cytokines involved in signaling pathways, including MK, PTN, SLIT, PDGF, WNT, BMP, and IGF. Notably, we observed significant downregulation of WNT and BMP signaling molecules in ectoderm-derived cells that communicate with mesoderm (Fig. 2i), which are critical for cartilage development. The findings above reveal that the absence of *Foxi3* in the ectoderm inhibits the proliferation of CNCCs and their lineages via cellular communication.

### FOXI3 in ectoderm and epidermis cells regulates CNCC-derived lineage development through chondrogenesis-related cytokines

**Fig. 3:**
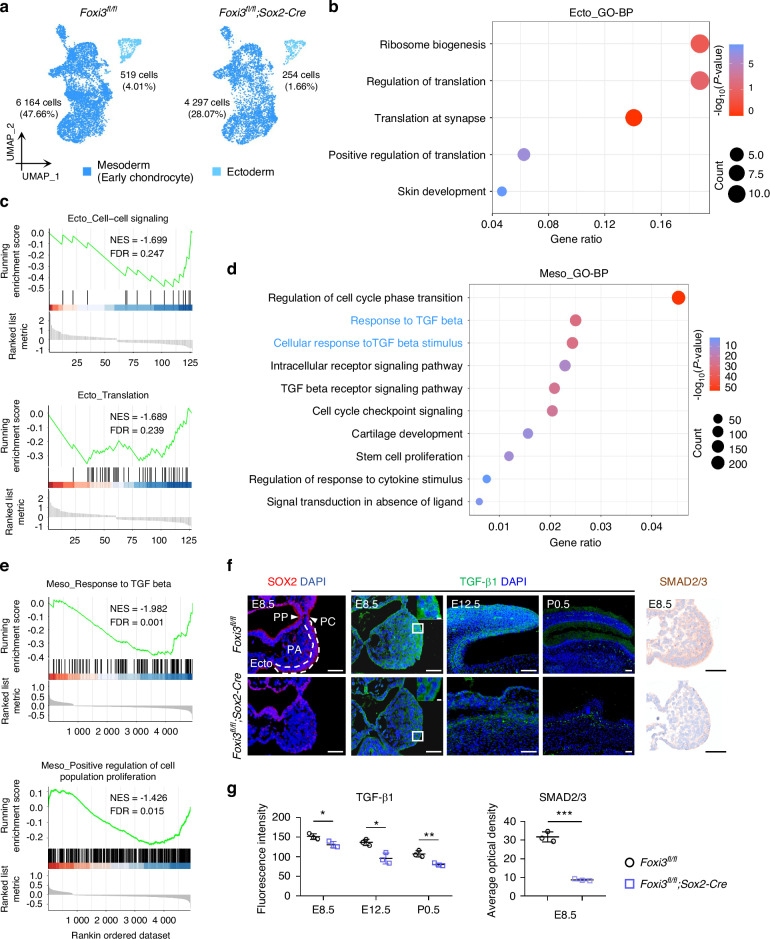
*Foxi3* in ectoderm and epidermal cells regulates the development of CNCCs and their lineages through cytokine signaling. **a** UMAP visualization of single cells from E10.5 *Foxi3*^fl/fl^; *Sox2-Cre* and control embryos. Percentages in parentheses represent the proportion of this cell subset relative to all cells in the pharyngeal arch (PA). **b** GO analysis of DEGs in the ectoderm of *Foxi3*^fl/fl^; *Sox2-Cre* and control embryos. Statistical significance was set at *P* < 0.05. **c** GSEA of DEGs showing downregulation of enriched GO-BP for cell-cell signaling (top panel) and translation (bottom panel) in the ectoderm cells of *Foxi3*^fl/fl^; *Sox2-Cre* mice. **d** GO analysis of DEGs in the mesoderm of *Foxi3*^fl/fl^; *Sox2-Cre* mice and control littermates (*P* < 0.05). **e** GSEA of DEGs showing downregulation of enriched GO-BP terms related to TGF-beta responses (top panel) and positive regulation of cell population proliferation (bottom panel) in mesoderm cells of *Foxi3*^fl/fl^; *Sox2-Cre* mice. **f** Immunofluorescence and immunohistochemistry staining demonstrate a significant decrease in TGF-β1 and SMAD2/3 expression in the PA or auricle in *Foxi3*^fl/fl^; *Sox2-Cre* mice compared to control littermates. Scale bars: 50 µm for low-power images and 10 µm for magnified views. **g** Quantitative fluorescence intensity analysis of TGF-β1 expression and average optical density analysis of SMAD2/3 are presented. n = 3 per genotype. ns no significance; *P <0.05, **P <0.01, and ***P <0.001 by unpaired two-tailed Student’s *t* test

To determine whether changes in FOXI3 expression in ectoderm cells influence the developmental environment of CNCCs and their lineage through chondrogenesis-related cytokine-mediated intercellular communication, we further analyzed the changes of ectodermal and mesodermal cells in the scRNA-seq data of the first and second pharyngeal arches from E10.5 *Foxi3*^fl/fl^; *Sox2*-Cre mice. The analysis revealed that the deletion of *Foxi3* in the ectoderm significantly reduced the number of ectodermal cells, while also affecting the number of mesodermal cells that do not express *Foxi3* (Fig. 3a). GO analysis of DEGs in the ectoderm cells indicated that FOXI3-regulated genes are related to translation and skin development (Fig. 3b). Additionally, gene set enrichment analysis (GSEA) demonstrated that both cell-cell signaling and translation pathways were downregulated (Fig. 3c). Significant effects were also observed in mesodermal cells, particularly in BPs related to cell cycle regulation, TGF-beta responses, intracellular receptor signaling pathways, cartilage development, stem cell proliferation, and responses to cytokine stimuli, as indicated by the GO analysis of DEGs (Fig. 3d). GSEA further revealed the downregulation of TGF-beta responses and a reduction in the positive regulation of cell population proliferation in the mesodermal cells (Fig. 3e). These findings underscore the influence of FOXI3 on mesodermal cell proliferation by facilitating communication between the ectoderm and mesoderm of the first and second pharyngeal arches, particularly through TGF-beta signaling. IF and immunohistochemistry (IHC) analysis confirmed these observations, showing a significant reduction in TGF-β1 and its downstream molecule SMAS2/3 expression in the pharyngeal arch and auricle of *Foxi3*^fl/fl^; *Sox2*-Cre mice at various developmental stages (Fig. 3f, g).

A cytokine array technique was subsequently employed to identify cytokines secreted by HaCaT cells. Twenty cytokines, including TGF-β1, were detected in the culture supernatant of HaCaT cells (Fig. S5a and Table S1). Kyoto Encyclopedia of Genes and Genomes (KEGG) enrichment analysis showed that these cytokines are involved in pathways related to chondrogenesis, including MAPK signaling,^38^ PI3K-Akt signaling,^39^ and focal adhesion^40^ (Fig. S5b). These observations suggested that epidermal cells may secrete cytokines capable of influencing chondrocyte proliferation. A four-dimensional label-free proteomics analysis of culture supernatants from FOXI3-knockdown HaCaT cells demonstrated a significant decrease in 266 secreted proteins and an increase in 76 proteins (Fig. S5c–e and Table S2). The downregulated differentially expressed proteins (DEPs) were enriched in BP related to TGF-β production (Fig. S5f).

To further investigate the effect of FOXI3-mediated communication between epidermal cells and chondrocytes, TGF-β1 levels were measured in HaCaT cells both intracellularly and extracellularly. Western blotting (WB) analysis was conducted on cell lysates (Fig. S5g), and enzyme-linked immunosorbent assay (ELISA) was performed on supernatants (Fig. S5h). The positive regulatory effect of FOXI3 on TGF-β1 was confirmed in vitro (Fig. S5g, h). Bulk RNA sequencing (RNA-seq) was carried out on C28/I2 cells co-cultured with FOXI3-knockdown HaCaT cells, resulting in the identification of 2489 downregulated and 2224 upregulated DEGs (Fig. S5i and Table S3). KEGG enrichment analysis revealed a marked attenuation of TGF-β signaling in chondrocytes (Fig. S5j). And compared to the control group, the levels of p-SMAD2/3 in C28/I2 cells were significantly reduced when co-cultured with FOXI3-knockdown HaCaT cells (Fig. S5k), further validating the attenuation of TGF-β signaling in chondrocytes. Enrichment of GO-BP terms in DGEs also included inhibition of the positive regulation of cell population proliferation (Fig. S5j). Furthermore, the introduction of a TGF-β pathway agonist (SRI-011381)^41^ into the co-culture medium successfully counteracted the inhibitory effect caused by FOXI3-knockdown HaCaT cells on the proliferation of MSC and C28/I2 cells (Fig. S5l). Furthermore, we administered the TGF-β signaling inhibitor SB-431542^42^ to wild-type mouse embryos (Fig. S5m) and found that it recapitulated the phenotype of a significant reduction in mandibular length similar to that in *Foxi3*^fl/fl^*;Sox2-Cre* mice (Fig. S5n–p). When *Foxi3*^fl/fl^*;Sox2-Cre* embryos were injected with the TGF-β pathway agonist SRI-011381, this intervention partially reversed the skeletal defects, demonstrating that activating the TGF-β pathway could rescue the phenotype caused by *Foxi3* deficiency (Fig. S5m, q). Overall, these findings provide evidence that FOXI3 in ectoderm and epidermal cells establishes a niche for CNCCs and their lineage cells, regulating their proliferation through cytokine-mediated communication, particularly via TGF-β1.

### FOXI3 regulates the translation and production of cytokines in epidermal cells

**Fig. 4:**
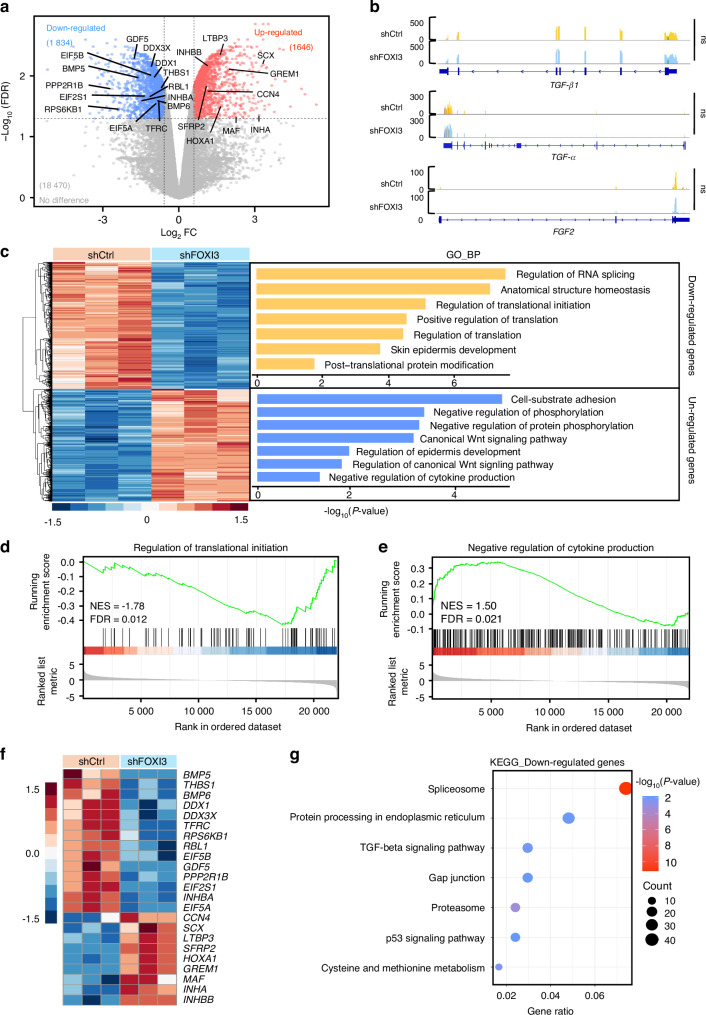
Identification and analysis of differentially expressed genes in FOXI3-knockdown HaCaT cells by bulk RNA sequencing. **a** Volcano plot of DEGs. DEGs with an FC >1.5 and FDR <0.05 are shown. **b** Visualization of TGF-β1, TGF-α, and FGF2 expression in RNA-seq using IGV software. No significant changes in mRNA levels were observed in FOXI3-knockdown HaCaT cells. **c** Heatmap (left) and GO analysis (right) of DEGs based on mRNA expression (red, upregulated; blue, downregulated). GSEA showing downregulated DEGs enriched in BP related to the regulation of translational initiation (**d**) and upregulated DEGs enriched in negative regulation of cytokine production (**e**). **f** Heatmap showing representative DEGs enriched in GO terms. **g** KEGG enrichment analysis of downregulated DEGs

To investigate whether FOXI3 directly regulates cytokine transcription, RNA-seq was performed on FOXI3-knockdown HaCaT cells to identify target genes of FOXI3. A total of 1 834 downregulated and 1 646 upregulated DEGs were identified (Fig. 4a and Table S4). However, the mRNA levels of key cytokines such as TGF-β1, TGF-β2, TGF-α, and FGF2 did not significantly change (Figs. 4b and S6a, b). GO enrichment analysis of BP showed that the downregulated DEGs were enriched in processes related to protein translation, including genes *DDX3X*, *DDX1*, *EIF5A*, *EIF2S1*, *EIF5B*, and *THBS1* (Fig. 4c, d, f). Conversely, the upregulated DEGs were enriched in negative regulation of cytokine production (e.g., *INHA*, *INHBB*) and protein phosphorylation (e.g., *INHA*, *GREM1*, *SFRP2*) (Fig. 4c, e, f). Notably, the downregulated DEGs were involved in pathways associated with the TGF-beta signaling pathway (Fig. 4g), reflecting reduced TGF-β levels in the culture environment. These findings suggest that FOXI3 exerts its influence on chondrogenesis-related cytokines not through direct transcriptional regulation, but by modulating their translation and production.

**Fig. 5:**
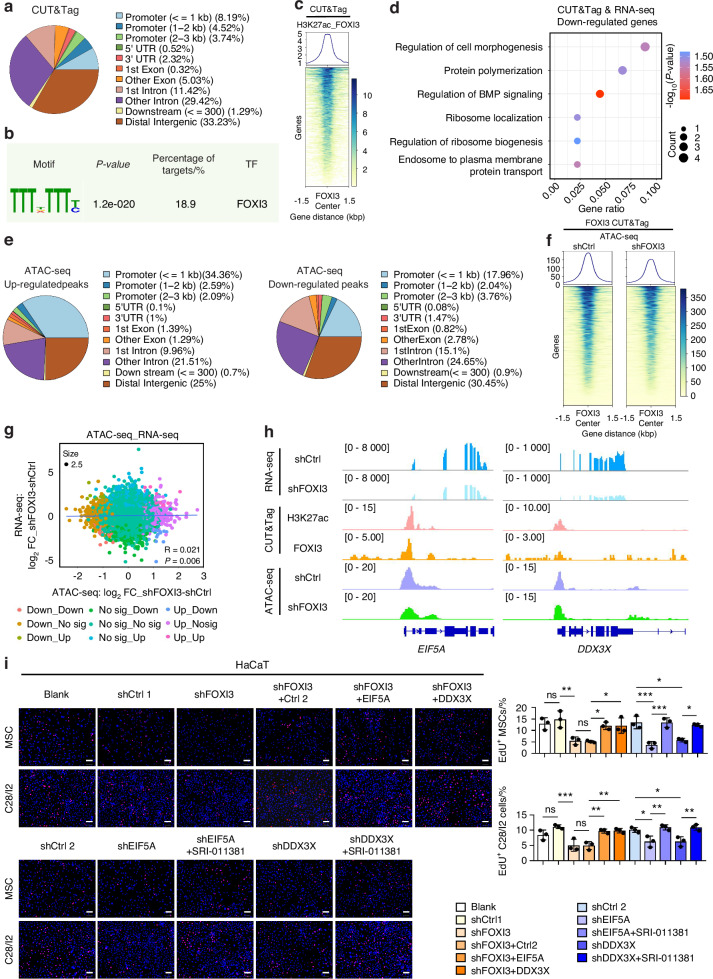
Genome-wide profile of FOXI3-binding genes and the open chromatin landscape in epidermal cells. **a** Genome-wide distribution of FOXI3-binding sites. **b** Annotated motif enriched at FOXI3-binding sites. **c** Heatmap showing acetylation states of FOXI3-bound regions in the genome. **d** GO analysis of downregulated target genes in FOXI3-knockdown HaCaT cells. **e** Genome-wide distribution of FOXI3-binding peaks with altered chromatin accessibility in FOXI3-knockdown HaCaT cells. **f** Heatmap showing chromatin accessibility changes in the FOXI3 CUT&Tag signal around genes in FOXI3-knockdown HaCaT cells. **g** Correlation of chromatin accessibility at all peaks and gene expression between control and FOXI3-knockdown HaCaT cells. **h** Normalized RNA-seq, CUT&Tag, and ATAC-seq profiles at the EIF5A and DDX3X loci. **i** Proliferation of MSCs and C28/I2 cells co-cultured with HaCaT cells was assessed using EdU (red) cell proliferation assays. Three replicates were conducted in each group. ns no significance; *P < 0.05, **P < 0.01, and ***P < 0.001 by one-way ANOVA with Tukey’s multiple comparison test

To identify FOXI3’s direct target genes, the cleavage under targets and tagmentation (CUT&Tag) technique was employed.^43^ A total of 1 544 peaks were revealed; most of these peaks were located in intergenic and intronic regions (Fig. 5a, Table S5). These peaks contained a putative FOXI3-binding motif (Fig. 5b), with high levels of H3K27 acetylation observed, indicating transcriptional activation (Fig. 5c). Genes with transcription factor-bound promoters were considered direct target genes.^44,45^ FOXI3 was found to occupy the promoter regions of 255 genes (Fig. 5a), including *CHD7* (the pathogenic gene associated with CHARGE syndrome, which presents with craniofacial defects^46^), *SF3B4* (whose heterozygous mutation leads to Nager syndrome, characterized by malformation of the craniofacial skeleton and the limbs^47^), and *PAX2* (the pathogenic gene for Papillorenal syndrome, which is characterized by abnormalities in the eyes, kidneys, and hearing^48^). However, none of them included chondrogenesis-related cytokines (Fig. S6c), suggesting indirect regulation of cytokine expression. GO and KEGG analyses of these target genes indicated significant enrichment in posttranscriptional regulation, cytokine biosynthesis, and metabolic processes (Fig. S7a, b). We then integrated the identified 255 target genes with RNA-seq data from FOXI3-knockdown HaCaT cells (compared to controls) by performing a differential expression analysis. This analysis identified 56 downregulated genes and 48 upregulated genes that were both bound by FOXI3 and transcriptionally altered upon FOXI3 loss. The downregulated genes were involved in protein polymerization and regulation of the BMP signaling pathway (Fig. 5d), while the upregulated genes were associated with anatomical structure homeostasis (Fig. S7c). These results, along with the effect of *Foxi3* deletion in mice on the protein expression levels of cytokines associated with cartilage development (Figs. 3f, g and S6d), collectively support our hypothesis that changes in FOXI3 expression in epidermal cells affect the expression of cytokines at the protein level by binding the promoters of genes related to translation.

Transcription factors often influence gene expression by modulating chromatin accessibility.^49^ To assess whether FOXI3 influences chromatin accessibility at its binding sites, an assay of transposase-accessible chromatin sequencing (ATAC-seq) was performed. A total of 2 229 regions, characterized by either lost (1 225) or gained (1 004) peaks, were identified in FOXI3-knockdown HaCaT cells compared to controls (Fig. S7d, Table S6). Lost peaks were found primarily in distal and intronic regions (Fig. 5e) and were associated with protein phosphorylation and the Wnt signaling pathway (Fig. S7e). Gained peaks were linked to cell-cell junction organization (Fig. S7f). Chromatin accessibility at FOXI3-binding sites was reduced upon FOX13 downregulation. A heatmap displayed a reduction in chromatin accessibility at FOXI3-binding sites upon FOXI3 downregulation (Fig. 5f), highlighting FOXI3’s role in modulating chromatin accessibility. However, a modest correlation was observed between changes in chromatin accessibility and gene expression in FOXI3-knockdown HaCaT cells, as indicated by the co-analysis of ATAC-seq and RNA-seq data (Fig. 5g).

Specific target genes were further investigated. Eukaryotic translation initiation factor 5A (EIF5A), a key player in translation elongation and termination,^50^ was identified as a primary target. Insufficient EIF5A levels can lead to translation errors and affect embryonic development.^51^ FOXI3 binds to the *EIF5A* promoter, and knockdown of FOXI3 reduced chromatin accessibility and *EIF5A* expression (Figs. 5h and S6a). Additionally, FOXI3 was found to bind the promoter of a DEAD-box helicase family member DDX3X (DBX, DDX3), an RNA helicase involved in RNA metabolism and cytokine production,^52–54^ with *DDX3X* expression also reduced upon FOXI3 knockdown (Figs. 5h and S6b). Quantitative real-time polymerase chain reaction (qPCR) analysis confirmed decreased expression of *EIF5A* and *DDX3X* in FOXI3-knockdown HaCaT cells, with no effect on cytokine mRNA levels (Fig. S6b). Additionally, reduced expression of EIF5A and DDX3X was observed in the pharyngeal arch ectoderm of the *Foxi3*^fl/fl^; *Sox2*-Cre mice at E8.5 (Fig. S6d). Meanwhile, overexpression of the two target genes rescued the inhibitory effect of FOXI3 downregulation on the proliferation of MSCs and C28/I2 cells in co-culture (Figs. 5i and S6e, f). Furthermore, when HaCaT cells with suppressed EIF5A or DDX3X were co-cultured, a reduction in MSC and C28/I2 cell proliferation was observed (Figs. 5i and S6g, h). This inhibitory effect was counteracted by the addition of a TGF-beta pathway agonist (Fig. 5i). These data indicate that FOXI3 controls the proliferation of CNCCs and lineage cells by modulating genes involved in the translation and production of cytokines in epidermal cells.

### Functional implications of a novel *FOXI3* frameshift mutation in craniofacial microsomia

**Fig. 6:**
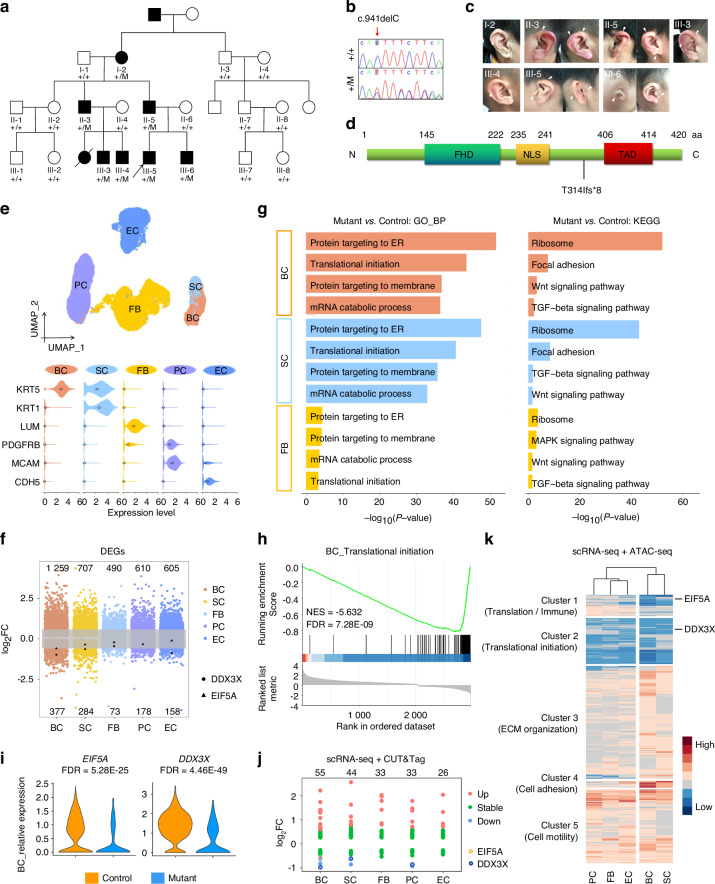
Single-cell RNA sequencing of a CFM patient auricle skin sample with a heterozygous *FOXI3* frameshift mutation. **a** Pedigree depicting individuals with the *FOXI3* mutation (c.941delC: p.T314Ifs*8). Filled symbols indicate affected members. “+,” wild-type allele; “M,” mutant allele. Some genotypes were undetermined due to unavailable DNA. The proband (arrow) provided auricular skin and cartilage for subsequent analysis. **b** Sanger sequencing of the *FOXI3* mutation site in healthy and affected individuals. Red arrow marks the mutation site. **c** Auricular deformities in *FOXI3*-mutant individuals. Arrows indicate affected areas. **d** FOXI3 protein diagram marking the mutation site within its functional domains. FHD, forkhead domain; NLS, nuclear localization sequence; TAD, transactivation domain. **e** UMAP visualization of single cells from control and mutant auricular skin. Violin plots display marker gene expression per cell type. Diamonds indicate medians. **f** Scatter plot of DEGs detected in each cell type of the control and mutant. BC basal cell, SC spinous cell, FB fibroblast, PC pericyte, EC endothelial cell. **g**, **h** Functional analyses reveal translational initiation downregulation in mutant BCs. **i** *EIF5A* and *DDX3X* expression decreased in mutant BCs. **j** Scatter plot of differentially expressed FOXI3 target genes, mapped from CUT&Tag data in HaCaT cells onto scRNA-seq profiles and analyzed across cell subtypes. **k** Heatmap displaying DEGs with altered chromatin accessibility in mutants versus controls. Genes exhibiting both differential expression (scRNA-seq) and promoter accessibility changes (ATAC-seq) were clustered into five groups based on enriched GO-BP terms. The color scale indicates relative expression (red, high; blue, low); rows represent genes, columns represent cell types, and the top dendrogram groups cell types by similarity

To validate the molecular mechanism of FOXI3, a pedigree marked by CFM carrying a novel heterozygous *FOXI3* frameshift mutation (c.941delC; p.T314Ifs*8) was examined (Fig. 6a).^55^ The mutation was identified through whole exome sequencing (WES), and co-segregation with the CFM phenotype was confirmed by Sanger sequencing within the pedigree (Figs. 6b, c and S8, and Table S7). The protein structure of FOXI3 includes a conserved DNA-binding forkhead domain (FHD), a nuclear localization sequence (NLS),^56,57^ and a C-terminal transactivation domain (TAD) (Fig. 6d).^19,55^ The frameshift mutation was found to result in protein truncation and loss of the TAD, which potentially reduced FOXI3 transcriptional activity (Fig. 6d). Pathogenicity analysis further underscored the deleterious impact of the mutation (Table S8).

To determine whether FOXI3 deficiency affects communication between epidermal cells and chondrocytes, scRNA-seq was performed on the auricle skin tissue of the patient (Figs. 6e and S9a). Five cell subtypes were identified based on marker genes (Figs. 6e and S9a–c). GO analysis of DEGs in patient samples revealed a pronounced influence of FOXI3 on genes associated with translational initiation in skin cells, particularly in BCs (Figs. 6f–h and S9d). This is also consistent with the fact that loss of *Foxi3* in mouse ectoderm has an impact on translation within ectoderm cells (Fig. 3b). KEGG enrichment analysis demonstrated the downregulation of ribosome and TGF-beta signaling pathways in the patient (Fig. 6f, g). *EIF5A* and *DDX3X*, both crucial regulators of translational initiation, were significantly downregulated in the patient’s BCs (Fig. 6f, i, j).

Additionally, differentiation impairments and a reduced capability of BCs to differentiate into other epidermal cell types were observed in the patient when compared to controls (Fig. S9e–h), which may contribute to the auricle skin deficiencies seen in CFM patients. This finding aligns with our previous observation that the deletion of *Foxi3* in the ectoderm impairs ectoderm and skin development in mice (Figs. 2c and 3a, b). By integrating scRNA-seq data from the patient with previous CUT&Tag results in HaCaT cells, it was found that differential expression of FOXI3 target genes was more pronounced in BCs compared to other skin cell types (Fig. 6j). Combining patient scRNA-seq data with previous ATAC-seq data from FOXI3 knockdown in HaCaT cells revealed altered chromatin accessibility in translation-related genes, particularly affecting BCs in the patient’s epidermal cells (Fig. 6k). These findings highlight the significant impact of the FOXI3 mutation on BCs and its role in regulating translational processes and cell differentiation in CFM.

**Fig. 7:**
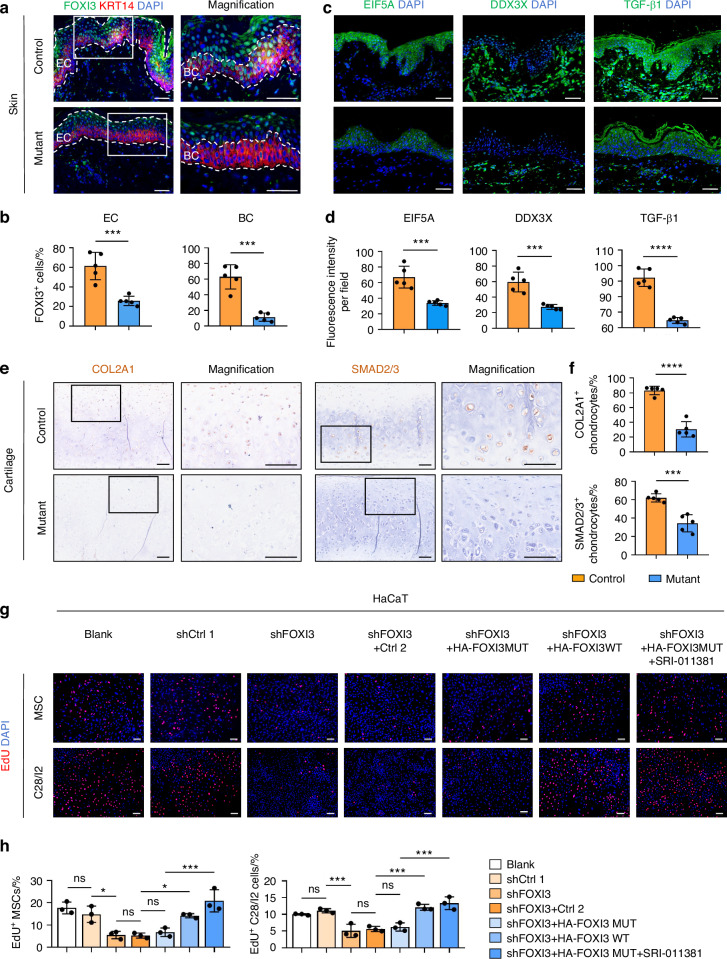
Confirmation of FOXI3’s role in a CFM patient sample and in an in vitro co-culture system. Immunofluorescence staining (**a**, **c**) and quantification (**b**, **d**) show reduced protein levels of FOXI3, EIF5A, DDX3X, and TGF-β1 in mutant auricular skin compared with controls. EC endothelial cell, BC basal cell. Immunohistochemistry staining (**e**) and quantification (**f**) demonstrate decreased expression of COL2A1 and SMAD2/3 in mutant auricular cartilage relative to controls. EdU proliferation assay (**g**) and quantification (**h**) of MSCs and C28/I2 cells co-cultured with HaCaT cells. Scale bars, 50 µm (**a**, **c**), 100 µm (**e**, **g**). Statistical significance was analyzed via unpaired two-tailed Student’s *t* test for (**b**, **d**, **f**), one-way ANOVA with Tukey’s multiple comparison test for (**h**). ns no significance; *P < 0.05, **P < 0.01, ***P < 0.001, and ****P < 0.000 1

Validation of FOXI3 protein expression in auricle skin and cartilage tissues from this patient was conducted using IHC and IF staining. A significant reduction in FOXI3 expression was observed in the patient’s auricle epidermal cells, especially in BCs, compared to the control group (Fig. 7a, b). A dramatic reduction in EIF5A and DDX3X expression was also revealed in the patient’s auricle skin (Fig. 7c, d). Interestingly, although no significant mRNA alterations were detected (Fig. S6a, b), a significant decrease in TGF-β1 protein was found in the patient’s auricle skin (Fig. 7c, d). Additionally, significant reductions in COL2A1^56^ and SMAD2/3,^57,58^ downstream genes of the TGF-beta signaling pathway, were observed in the patient’s auricle cartilage (Fig. 7e, f).

Further investigation into the functional consequences was conducted by examining the effects of FOXI3 downregulation in HaCaT cells, MSCs, and C28/I2 cell proliferation during co-culture. Overexpression of the FOXI3 mutant failed to rescue the inhibitory effect, unlike wild-type FOXI3 (Figs. 7g, h and S2i). However, administering a TGF-β agonist in the mutant overexpression system restored this phenotype (Fig. 7g, h).

Taken together, these findings provide compelling evidence for FOXI3’s involvement in auricle cartilage development by regulating translation and promoting cytokine production associated with chondrogenesis in epidermal cells.

## Discussion

The role of FOXI3 as a transcription factor in organ development and its association with CFM have been well established, yet the precise molecular mechanisms underlying its function remain unclear. This study addresses the discrepancy between FOXI3’s expression in the ectoderm and auricle epidermis and its involvement in CNCC and cartilage development. Previous studies documented that FOXI3 is exclusively expressed in the ectoderm and endoderm during embryogenesis, with limited expression in the mesoderm or CNCCs that make up the mesenchyme of the first and second pharyngeal arches.^12,17,18,59,60^ However, the potential for transient FOXI3 expression in CNCCs, which may have significant implications for cell fate determination, has likely been overlooked. To address this, CNCC-specific *Foxi3* knockout mice were generated, showing no CNCC development abnormalities and excluding transient FOXI3 expression as a factor. Previous studies have shown no increase in CNCC apoptosis in *Foxi3*^−/−^ mice before E10, which is consistent with our observations in E8.5 *Foxi3*^fl/fl^; *Sox2-Cre* mice. Although *Foxi3* deletion in mice does not impact early CNCC migration from the neural tube,^12^ a decrease in CNCC proliferation was observed at E8.5, potentially due to the absence of *Foxi3* in the niche of CNCCs provided by pharyngeal arch ectoderm.

Intercellular communication between neighboring cells plays a pivotal role in influencing growth behavior through cellular signaling pathways.^61^ The deletion of *Foxi3* in the ectoderm resulted in reduced pharyngeal arch size, disrupted ectodermal structure, and impaired CNCC proliferation. Additionally, the TGF-beta signaling in mesoderm cells was diminished. For the first time, single-cell profiles of the first and second pharyngeal arches in mice were provided, proposing a novel model in which FOXI3 in ectoderm cells facilitates CNCC proliferation through cytokine-mediated cell-to-cell communication.

The deletion of *Foxi3* in the ectoderm was also found to impair cellular translation processes. We suggest that FOXI3 influences cytokine production by regulating translation-associated genes rather than directly modulating cytokine transcription. FOXI3’s unique expression patterns suggest that its influence may extend beyond the mandible and auricle. ScRNA-seq analysis of auricle skin from a CFM patient with a heterozygous *FOXI3* frameshift mutation corroborated the findings, revealing an uncharted mechanism that highlights the diverse functional roles of FOXI3. The role of FOXI3 may extend to organs where it is expressed in their epithelial layers, such as the tooth, glottis, epiglottis, parathyroid, and thymus.^61^ These organs could also utilize similar intercellular communication mechanisms by FOXI3 to support proper development and function. This highlights FOXI3’s broader impact on regulating cell-to-cell communication and various cellular processes across tissues and organs.

Previous studies have linked FOXI3 to FGF signaling during pharyngeal development and CNCC survival through the regulation of *Fgf8* transcription.^12^ However, this study did not observe direct regulation of *FGF8* by FOXI3 (Fig. S10a), nor did it detect significant effects on FGF signaling pathways in either FOXI3 knockout mouse models^62,63^ or in auricle skin samples from our CFM patient with a heterozygous *FOXI3* frameshift mutation (Fig. S10b). The discrepancy in findings suggests that FOXI3 may indirectly regulate genes that affect FGF8 through translation or other functions, warranting further investigation into the FOXI3-FGF8 relationship.

To uncover FOXI3’s direct target genes, CUT&Tag technology was used to identify FOXI3-binding sites in epidermal cells. Singh et al. performed a Chromatin immunoprecipitation (ChIP) assay with anti-FLAG M2 magnetic beads to explore FOXI3 bindings sites and its role as a transcriptional activator on the *AE4* promoter.^64^ In this study, FOXI3 antibodies were rigorously evaluated to identify one suitable for CUT&Tag, which allowed the generation of the first genome-wide endogenous FOXI3 occupancy data. The analysis identified *EIF5A* and *DDX3X* as direct FOXI3 target genes, both involved in translation regulation and crucial for embryonic development.^65,66^ EIF5A is expressed during key stages of mouse embryonic development, particularly in auricle formation,^66^ and plays a critical role in cytokine secretion and signaling.^67,68^ DDX3X, an RNA helicase, has been implicated in regulating TGF-β1 translation and MSC differentiation into chondrocytes.^69^ TGF-β1 synthesis is independently regulated at translation and secretory levels in response to specific stimuli.^70^ These findings explain how FOXI3 influences organ development by regulating cytokine translation and secretion through its direct targets, EIF5A and DDX3X.

It is important to note that *FOXI3* expression is nearly undetectable in RNA-seq (Fig. S11a) and scRNA-seq (Fig. S11b) data. This observation suggests that the high GC content (67%) of *FOXI3* cDNA may interfere with its amplification (Fig. S11c), due to current technological limitations. However, *FOXI3* expression was successfully detected using qPCR, aided by strategic primer design to avoid regions of elevated GC content.

In conclusion, this study underscores FOXI3’s critical role in the ectoderm niche for proper CNCC development. By regulating cytokine translation and production, FOXI3 enables the essential cell-to-cell communication necessary for CNCC and cartilage development. These insights broaden the understanding of FOXI3’s contributions to both development and disease.

## Materials and methods

### Patients and sample collection

The auricle sample from a 13-week gestation human fetus was voluntarily obtained after the donor couples provided informed consent from the affiliated hospital of Nantong University. Additional auricle skin and cartilage samples were procured from discarded tissues during various surgical procedures. All tissues were preserved in RNAlater (Beyotime Biotechnology, Shanghai, China) for RNA extraction, in 4% paraformaldehyde (PFA) for IHC and IF staining, or in MACS Tissue Storage Solution (Miltenyi, Germany) for scRNA-seq. Specific sample details for scRNA-seq are provided in Table S9.

Discarded auricle cartilage and skin tissues from the proband were collected during right auricle reconstruction surgery, fixed in 4% PFA, and embedded in paraffin for subsequent IF staining. During left auricle corrective surgery, auricle skin tissue was harvested for scRNA-seq, though no surplus cartilage tissue was available for analysis.

This study was reviewed and approved by the Institutional Research Ethics Committee of the affiliated hospital of Nantong University (2020-K013) and the Eye & ENT Hospital of Fudan University (2020069).

### Genetic analysis

The *FOXI3* mutation detected in WES data was characterized using DbSNP and the 1000 Genomes Project. Conservation of the mutant site was analyzed with Polyphen-2 (http://genetics.bwh.harvard.edu/pph2/), while the effects of protein-altering mutations on structure and function were predicted using SIFT (http://sift.jcvi.org/) and PROVEAN (https://www.jcvi.org/research/provean) (Table S8). The mutation was confirmed in the CFM pedigree by extracting total DNA from blood samples using QIAamp DNA Blood Mini Kit (Qiagen, Hilden, NRW, Germany), amplifying via PCR, and sequencing using the Sanger (Fig. S8).

### Immunohistochemistry and immunofluorescence

Tissues preserved in 4% PFA were paraffin-embedded and sectioned. After hydration in graded ethanol (100%–50%), sections were incubated with 3% H_2_O_2_ for 10 min. Sections were then blocked with goat serum (Sigma-Aldrich) and incubated overnight at 4 °C with primary antibodies against FOXI3 (1:100, Bioss, Beijing, China), COL2A1 (1:100, Abmart, Shanghai, China), and SMAD2/3 (1:100, Abmart). The following day, sections were treated with a secondary antibody and an ultrasensitive DAB kit (Sigma-Aldrich). Images were captured using a light microscope (Leica), and quantitative analysis of positive brown cells was performed using ImageJ software (v.1.52) across five equal magnification fields per sample.

For immunofluorescence, sections were similarly blocked with goat serum and incubated overnight at 4 °C with primary antibodies against SOX2 (1:100, Abcam, Cambridge, MA, USA), KRT14 (1:100, Abmart), TGF-β1 (1:100, Abmart), DDX3X (1:50, Abmart), EIF5A (1:100, Abmart), Ki67 (1:100, Abmart), BMP4 (1:100, Abmart), FGF2 (1:100, Abmart), and FGF7 (1:100, Abmart). The next day, sections were incubated with fluorescent secondary antibodies (Yeasen Biotech) and imaged using a fluorescence microscope (Leica).

### RNAscope in situ hybridization and imaging

Fresh mouse embryos were cryosectioned to a thickness of 6 μm and subsequently mounted on Superfrost Plus microscope slides (Thermo Fisher Scientific, Waltham, MA, USA). In situ hybridization was conducted using the RNAscope® Multiplex Fluorescent Detection Reagent v2 (Cat. No. 323110; Advanced Cell Diagnostics, Newark, CA, USA) to enable the detection of target mRNAs at the single-cell level. For this study, the custom-designed mouse RNAscope® Probe-Mm-FOXI3-C2 (Cat. No. 490631-C2; Advanced Cell Diagnostics, Newark, CA, USA) was obtained from the manufacturer. All experimental procedures were performed in strict accordance with the manufacturer’s instructions to ensure consistency and reliability of results. To visualize cell nuclei, samples were counterstained with DAPI. To validate sample mRNA integrity and to rule out non-specific labeling, control probes were incorporated into the experimental design. High-resolution images of the labeled samples were captured using an Akoya PhenoImager HT scanner (Akoya Biosciences, Marlborough, MA, USA).

### Mice ethical approval and generation of *Sox2* lineage-tracing mice

All procedures involving mice were approved by the Institutional Animal Care and Use Committee at Fudan University (SYXK2020-0032). The *Sox2-Cre* mice were kindly provided by Prof. Ouyang K (Peking University Shenzhen Hospital), while the *Rosa26-lsl-tdTomato* mice were purchased from the Shanghai Model Organisms Center, Inc. For lineage-tracing experiments, *Sox2-Cre; Rosa26-lsl-tdTomato* mice were generated by crossing heterozygous *Sox2-Cre* mice with heterozygous *Rosa26-lsl-tdTomato* mice. All mice were on a C57BL/6 background.

### Whole-embryo tissue clearing and 3D light-sheet imaging

E10.5 mouse embryos were fixed in 4% PFA at 4 °C for 24 h. After washing with PBS, the embryos were transferred to CUBIC reagent-1 and incubated at 37 °C for 5 days. Following another wash PBS, the embryos were placed in a 20% sucrose solution at room temperature for 1 day. The embryos were then transferred to CUBIC reagent-2 and stored overnight at room temperature. Subsequently, the samples were incubated in a 37 °C water bath for 6 days. Imaging was performed using a two-photon microscope system (A1R MP+, Nikon, Tokyo, Japan) with CUBIC reagent-2 as the imaging medium. The system was equipped with a 16× water immersion objective (NA 0.8) and utilized a 1020-nm laser for excitation, along with a 629 ± 56-nm bandpass filter for tdTomato fluorescence. Z-stack images were converted to 16-bit TIFF format with ImageJ (v.1.52) and were maximally projected in 3D using Zen software (v.3.9)^71^.

### Generation of *Foxi3* conditional knockout mice

The *Foxi3*^*fl/+*^ mice (C57BL/6J background) were generated using ESC (C57BL/6N background) targeting technology by the Shanghai Model Organisms Center, Inc., LoxP sites flanking exon 2 via homologous recombination. To delete *Foxi3* in CNCCs, *Foxi3*^*fl/+*^ mice were crossed with *Wnt1-Cre* mice,^72^ and for ectodermal deletion, they were crossed with *Sox2-Cre* mice.^35^ Genotyping primers are listed in Table S10. *Foxi3* knockout efficiency was confirmed via IF. Embryonic stages were defined with the morning of the vaginal plug as E0.5. Transgenic mice were backcrossed with wild-type C57BL/6J mice after every three generations. All experiments were approved by the Experimental Animal Ethics Committee of the Eye & ENT Hospital of Fudan University (IACUC-DWZX-2023).

### Micro-computed tomography

The heads of 4-week-old mice were scanned using a micro-CT system (μCT-80, Scanco Medical AG, Bassersdorf, Switzerland) in high-resolution scanning mode (voxel size and slice thickness: 20 μm). The external and middle ear structures were observed in cross-sectional images.

### Intraperitoneal injection in pregnant mice and phenotype observation

Pregnant mice at 7.5 days post-fertilization were injected intraperitoneally with SB-431542 (10 mg/kg in 5% DMSO) or SRI-011381 (30 mg/kg in 5% DMSO) for 5 consecutive days. The craniofacial phenotype of the mice was observed at P1 or E14.5.

### Skeletal staining

Newborn mice were euthanized, stored in PBS on ice, and then scalded in hot tap water (70 °C) for approximately 15 s. The skin was carefully peeled off, and all internal organs were removed. The bodies were fixed in 95% ethanol for 24 h and stained with Alizarin Red and Alcian Blue as described in previous studies,^4^ and photographed using bright-field microscopy.

### Cell culture

HaCaT and C28/I2 cells were cultured in Dulbecco’s modified Eagle’s medium (DMEM) (Biological Industries, Beit HaEmek, Israel) with 10% fetal bovine serum (FBS) (Gibco, Carlsbad, CA, USA) and 1% penicillin-streptomycin (NCM Biotech, Suzhou, China) at 37 °C in 5% CO_2_.

MSCs were cultured in DMEM/F12 (Gibco) with 10% FBS (Gibco) and 1% penicillin-streptomycin (NCM Biotech) at 37 °C in 5% CO_2_. Cells at passages 3–7 were used for experiments.

### Plasmid construction

The plasmids FOXI3-pCDH (pCDH-CMV-MCS-EF1-Puro), FOXI3-c.941del-pCDH, EIF5A-pCDH, and DDX3X-pCDH were obtained from GeneRay (Shanghai, China) and confirmed by sequencing. The empty vector was used as a negative control. Plasmids were amplified, extracted, and concentrated using the GML-PC Lentivirus Concentration Kit (Genomeditech, Shanghai, China). HaCaT cells were transfected with these plasmids or the empty vector using HitransG P (Genechem, Shanghai, China) and screened with puromycin 48 h post-transfection. Overexpression efficiency was measured by WB or qPCR. Short hairpin RNA (shRNA) molecules targeting the coding sequences of human *FOXI3*, *EIF5A*, and *DDX3X* were designed by Genechem. The shRNA targeting *FOXI3* was cloned into the GV112 vector, and the shRNA sequences targeting *EIF5A* and *DDX3X* were cloned into the GV248 vector. The shRNA constructs were transfected into HaCaT cells, with cells transfected with empty vectors serving as negative controls. Two days post-transfection, cells were seeded at very low density and selected with puromycin. Knockdown efficiency was assessed by qPCR.

### Co-cultivation system construction

MSCs or C28/I2 cells were seeded into a 24-well plate for transwell co-culturing. The following day, a 0.4 μm-pore size chamber (Corning Costar, Cambridge, MA, USA) containing HaCaT cells was placed into the 24-well plate. Both cell types were cultured in a shared medium.

### Cell proliferation assay

DNA replication activity was measured using the Cell-Light EdU Apollo567 In Vitro Kit (Ribobio, Guangzhou, China) according to the manufacturer’s protocol. After 3 days of co-culture, 50 μmol/L EdU was added to the medium of the lower chamber, which contained MSCs and C28/I2 cells. Cells were incubated at 37 °C for 2 h, and images were acquired with a fluorescence microscope (Leica) and quantified using ImageJ software (v.1.52).

### Cell apoptosis measurement

Apoptosis was analyzed using the Annexin V-Alexa Fluor 647/PI Apoptosis Detection Kit (Yeasen Biotech). MSCs and C28/I2 cells were collected from 6-well subplates after 3 days of co-culture with HaCaT cells. The cells were resuspended in 200 μL of binding buffer, labeled with Alexa Fluor 647 and PI for 15 min at room temperature, and analyzed by flow cytometer (BD, Franklin Lakes, NJ, USA). Data were processed using FlowJo software (v.10.8).

### Quantitative real-time polymerase chain reaction

Total RNA was extracted using TRIzol reagent (Invitrogen). RNA was reverse-transcribed using the RT Reagent Kit (Takara, Otsu, Shiga, Japan), and real-time qPCR was performed using the ABI Step One Plus Real-Time PCR System (Carlsbad, CA, USA) with TB Green^®^ Fast qPCR Mix (Takara), following the manufacturer’s instructions. Primer sequences used for qPCR are listed in Table S10.

### Western blotting

Cells were lysed using radioimmunoprecipitation assay buffer (Yeasen) containing a protease inhibitor cocktail (Sigma-Aldrich). Protein concentration was determined using the Bradford Protein Assay Kit (Abcam). Samples were subjected to gel electrophoresis (NCM Biotech) and transferred onto nitrocellulose membranes (Pall, Port Washington, NY, United States). The membranes were blocked with 8% milk in Tris-buffered saline Tween (TBST) for 1 h at room temperature, followed by overnight incubation at 4 °C with primary antibodies. After incubation with secondary antibodies at room temperature for 1 h, protein bands were detected. The following antibodies were used: anti-HA (1:5 000, Abcam), anti-EIF5A (1:5 000, Abmart), anti-DDX3X (1:1 000, Abmart), anti-VCL (1:5 000, Abmart), anti-GAPDH (1:5 000, ProteinTech, Wuhan, China), anti-TGF-β1 (1:1 000, Abmart), SMAD2/3 (1:500, Affinity Biosciences, Melbourne, Australia), p-SMAD2 (1:1 000, Cell Signaling Technology, Boston, USA), p-SMAD3 (1:1 000, Cell Signaling Technology), and anti-TGF-β1 (1:1 000, Cell Signaling Technology).

### Single-cell RNA sequencing

Human skin tissues were incubated at 37 °C for 1 h in PBS containing 2 mg/mL collagenase I, 2 mg/mL collagenase IV, 2 mg/mL dispase, and 0.125% trypsin-EDTA to generate single-cell suspensions. Cartilage samples were digested in 0.2% collagenase Ⅱ at 37 °C for 2 h, with cells collected every 30 min until 14 h. Pharyngeal arches from E10.5 homozygous *Foxi3*^fl/fl^; *Sox2-Cre* embryos (*n* = 4) and control littermates (*n* = 26), as well as the entire auricles of E15.5 and P7 wild-type mice, were digested and separated into single-cell suspensions (CapitalBio Technology, Beijing). cDNA was synthesized and amplified for scRNA-seq library construction, followed by sequencing on the Novaseq6000 platform (Illumina, USA).

### Analysis of single-cell RNA sequencing data

Digital gene expression matrices were generated using the Cell Ranger pipeline with human reference GRCh38 and mouse reference GRCm39. The matrices were analyzed using R (v.4.0.0) and the Seurat package (v.4.2.0). Low-quality cells were excluded based on the following criteria: (1) fewer than 800 unique molecular identifiers (UMIs), (2) more than 6 000 or fewer than 800 genes, and (3) greater than 15% of UMIs derived from mitochondrial genes. Gene expression matrices were normalized using the NormalizeData function, and the top 2 000 highly variable genes (HVGs) were identified using the FindVariableFeatures function. The RunPCA function was applied to scaled data produced by the ScaleData function to reduce dataset size. Batch effects were eliminated using the RunHarmony function from the harmony package (v.1.0), and the top 15 harmony components were used for subsequent analysis with the RunUMAP function. Cell clustering was performed using the FindNeighbors and FindClusters functions with a resolution parameter of 0.5, based on the first 15 harmony components. Marker genes for each cluster were identified using the Wilcoxon rank-sum test via the FindAllMarkers function.

Cell type-specific DEGs were identified using the FindAllMarkers function with default parameters, considering only genes expressed in at least 10% of cells within a cluster with a Log_2_ fold change (Log_2_FC) > 0.5 and Bonferroni-corrected *P* <0.05 as significant DEGs. Biological functions enriched for DEGs were analyzed using the enrichGO function from the clusterProfiler package (version 3.18.1), with enriched GO terms filtered based on adjusted *P*-values (Benjamini-Hochberg-adjusted *P* <0.05).^73^ Ligand-receptor interactions were identified using the CellPhoneDB package^74^ (v.2.1.2), with interactions considered significant if *P* <0.05 after 1 000 random permutations. Network analysis to compute centrality scores of outgoing, incoming, and mediator communication pathways was conducted using CellChat (v1.6.0). The identification of dominant and most relevant pathways was achieved by manually exploring and comparing the network results, in addition to utilizing the visualization tools provided by CellChat for scDiffCom results.

Pseudotemporal ordering of human BCs was performed using the Monocle 2 package,^75^ identifying DEGs across different development conditions using the differentialGeneTest function. Genes with a *Q*-value <0.01 were included in the construction of the pseudotime trajectory. CytoTRACE analysis under default parameters^76^ was also performed to predict differentiation states based on transcriptional diversity, complementing the Monocle trajectory analysis.

### Cytokine array detection

Supernatants from wild-type HaCaT cells and untreated complete medium were sent to RayBiotech, Inc. (Guangzhou, China) for cytokine array detection, which included 41 detection indexes. The raw data were background-subtracted and normalized using Raybiotech software. DEGs were identified using the following conditions: (1) |log_2_FC| >1.2 and (2) mean signal value (fluorescence) >150.

### 4D-label-free quantification proteomics

Supernatants from FOXI3-knockdown HaCaT cells and control cells were collected for 4D-label-free quantification proteomics, conducted by APTBIO Co, Ltd. (Shanghai, China). Protein concentrations were measured using the BCA Protein Assay Kit (Bio-Rad, USA). Protein digestion with trypsin was performed based on the filter-aided sample preparation procedure. Proteins were separated on 12.5% SDS-PAGE gel (NCM Biotech) and visualized using Coomassie Blue R-250 staining (Yeasen). LC-MS/MS analysis was performed using a timsTOF Pro mass spectrometer (Bruker Daltonics, Billerica, MA, USA) coupled with Nanoelute (Bruker Daltonics). Raw MS data were combined and processed in MaxQuant software (v.1.5.3.17) for identification and quantitation analysis. Significantly DEPs were identified based on the following: (1) FC >2 and *P* <0.05 or (2) protein detected at least twice in one sample but absent in the other.

### RNA sequencing

Total RNA was extracted from the cells using TRIzol reagent (Invitrogen), with RNA quantity and purity assessed via Bioanalyzer 2100 and RNA 6000 Nano LabChip Kit (Agilent). Only samples with an RNA integrity number (RIN) >7.0 were used for library construction. Paired-end (PE) RNA sequencing was performed on the Illumina Novaseq TM 6000 platform (Illumina, San Diego, CA, USA). PE reads were filtered using Cutadapt (v.1.9), and quality checks were performed using FastQC (v.0.11.9). The mapped reads of each sample were assembled with StringTie (v.2.1.6) under default parameters, and the transcriptomes were merged using gffcompare (v.0.9.8). Transcript abundance was estimated using Ballgown (http://www.bioconductor.org/packages/release/bioc/html/ballgown.html) and StringTie to calculate fragment per kilobase of transcript per million mapped reads the value. Differential gene expression analysis was conducted with DESeq2 (v.1.30.0) under default settings. DEGs were defined as genes with a false discovery rate (FDR) <0.05 and FC >1.5. All experiments were conducted with three biological replicates.

### Cleavage under targets and tagmentation

CUT&Tag was conducted following the manufacturer’s instructions (Vazyme Biotech, Nanjing, China). Approximately 100 000 cells were harvested and washed with 1 mL of Wash Buffer at room temperature, followed by centrifugation at 600 × *g* for 3 min. The cells were resuspended in 100 μL of Wash Buffer and rotated with 10 μL ConA beads. Cells were incubated with anti-FOXI3 (Origene, Rockville, MD, USA) diluted at 1:50 and anti-H3K27ac (Abcam) diluted at 1:100 in an antibody buffer for 2 h at room temperature. Permeabilized nuclei were centrifuged and incubated with a 1:100 dilution of anti-rabbit IgG antibody (Vazyme Biotech) in 100 μL of Dig-wash Buffer for 1 h at room temperature. Following two washes with Dig-wash Buffer, nuclei were incubated with a 1:100 dilution of pG-Tn5 in Dig-300 Buffer for 1 h at room temperature on a rotator. Cells were washed three times with Dig-300 Buffer, resuspended in 300 µL of Tagmentation Buffer, and incubated at 37 °C for 1 h. Tagmentation was stopped by adding Stop Buffer (100 μL Buffer L/B, 20 μL DNA Extract Beads, and 5 μL 20 mg/mL Proteinase K). Samples were kept for 10 min at 55 °C in an incubator. The remaining steps were carried out according to the product manual. Samples were amplified with Illumina primers, and libraries were purified using a QiaQuick PCR purification kit (QIAGEN) with size selection performed using 0.7× and 0.2× DNA clear beads (TransNGS). Libraries were sequenced via paired-end 150 bp reads on a NovaSeq platform (Anoroad).

### Analysis of cleavage under targets and tagmentation data

Adapter sequences and low-quality reads were removed using Fastp (v.0.20.0) with parameters “-thread 8 -5 -3 -W 4.” Paired-end reads were aligned using Bowtie2 (v.2.3.5.1) with the parameter “-p 8, -sensitive.” Duplicated reads were filtered with Picard (v.2.22.8) using “REMOVE_DUPLICATES=true.” Fragment ratio in peaks (FRIP) values were calculated using bedtools (v.2.29.2) and awk (v.4.0.2). A bigWig file was generated using deepTools for RPKM normalization and visualized in IGV. Peak calling was performed with HOMER, using the findPeaks program with the “-style histone” option. The enriched peak regions from HOMER were used as input for DESeq2 (v.1.30.0) to find differential peaks. Motif analysis was conducted using MEME Suite (https://meme-suite.org/meme/tools/meme).

### Assay of transposase-accessible chromatin sequencing

To profile open chromatin regions, 50 000 control and FOXI3-knockdown HaCaT cells were washed once with cold PBS. Cells were lysed on ice for 3 min in 50 μL of ice-cold Lysis Buffer (10 mmol/L Tris, pH 7.4, 10 mmol/L NaCl, 3 mmol/L MgCl_2_, 0.1% NP-40, 0.1% TWEEN 20, 0.01% Digitonin in DEPC H_2_O), resuspended in ice-cold RBS-Wash (10 mmol/L Tris, pH 7.4, 10 mmol/L NaCl, 3 mmol/L MgCl_2_, 0.1% TWEEN 20) and pelleted at 4 °C at 500 × *g* for 10 min. Tagmentation was conducted in 1× Tagmentation Buffer (10 mmol/L Tris, pH 7.4, 5 mmol/L MgCl_2_, 10% DMF, 33% PBS, 0.1% TWEEN 20, 0.01% Digitonin) using 100 nmol/L Tn5 Transposase for 15 min at 37 °C. The same volume of 2x Tn5 Digestion Mix (TransNGS, Beijing, China) was added. Tagmentated samples were purified with a QIAquick PCR Purification Kit (QIAGEN). Libraries were amplified with Illumina primers, purified, and size selected using 0.7× and 0.2× DNA clear beads (TransNGS). The ATAC-seq libraries were sequenced using paired-end 150 bp reads on a NovaSeq platform (Anoroad, Beijing, China).

### Analysis of assay of transposase-accessible chromatin sequencing data

Fastp (v.0.20.0) was used to remove adapter sequences and low-quality reads. The remaining reads were aligned to the human genome (hg38) using Bowtie2 (v.2.3.5.1) with the parameters “-sensitive, -X 2000.” PCR duplicates were filtered with Picard (v.2.22.8). FRIP values were calculated with bedtools (v.2.29.2) and awk (v.4.0.2). deepTools (v.3.5.1) was used to generate a bigWig file with reads per kilobase per million normalization for visualization in IGV (v.2.14.1). SAM files were converted to BAM format using Samtools (v.0.1.19), and peak calling was performed with MACS2 (v.2.2.4) using the parameters “-t input_file –q 0.01 -f BAM -nomodel -shift −100 -extsize 200 -keep-dup all.” Differentially enriched regions between experimental and control groups were identified using DiffBind (v.2.14.0), and differential genes were determined using edgeR and DESeq2.

## Supplementary information

Supplemental Tables

Supplemental Figure 1

Supplemental Figure 2

Supplemental Figure 3

Supplemental Figure 4

Supplemental Figure 5

Supplemental Figure 6

Supplemental Figure 7

Supplemental Figure 8

Supplemental Figure 9

Supplemental Figure 10

Supplemental Figure 11

Supplemental Figure Legends

## Data Availability

The data underlying this article are available in the article, and CNCB (raw sequencing data) at https://www.cncb.ac.cn/ (CNCB accession: PRJCA020077, 019727, and 019720), and on the UCSC genome browser session is https://genome.ucsc.edu/s/Siyi%20Wu/HaCaT%20cell%20data.
