# Supplementary figures and images for: FOXI3 establishes the ectodermal niche in pharyngeal arches for cranial neural crest cells and their lineages
Source: Bone Res. 2026 Feb 4;14:16. doi: 10.1038/s41413-025-00499-w (PMC12873258; doi:10.1038/s41413-025-00499-w)

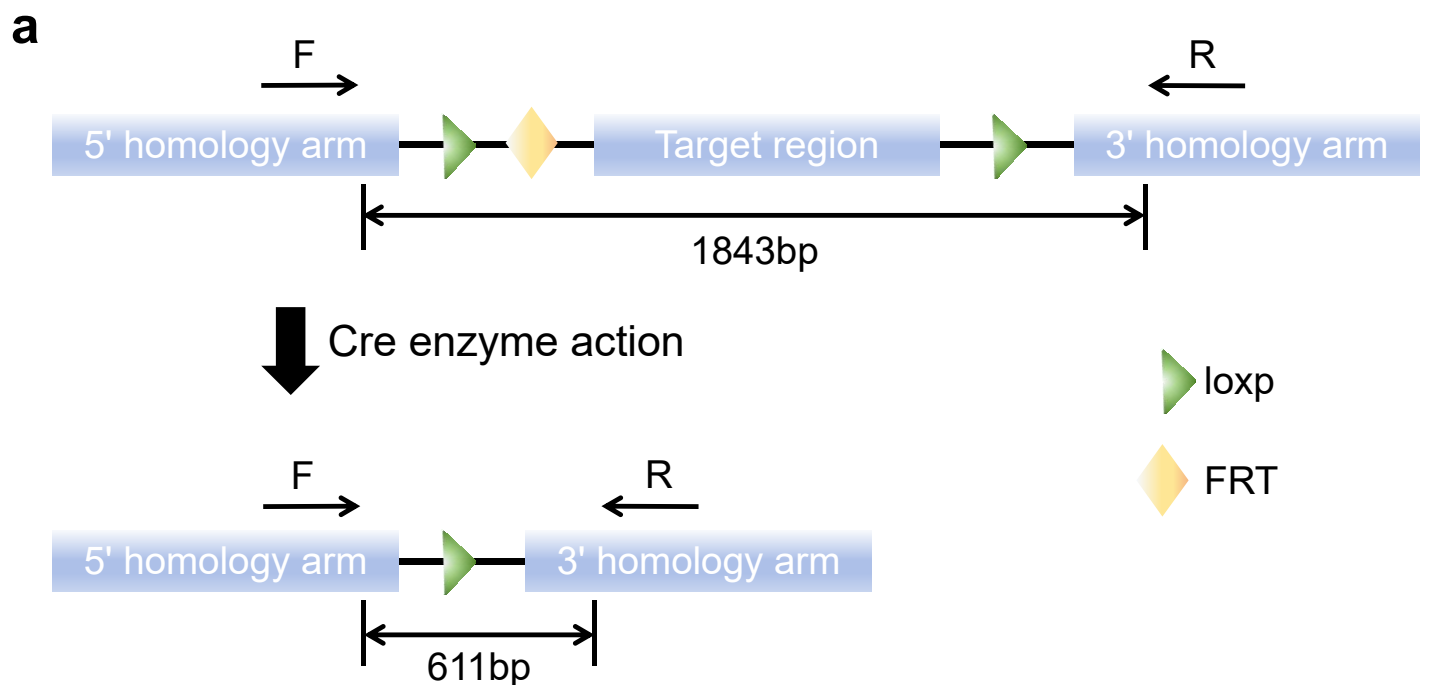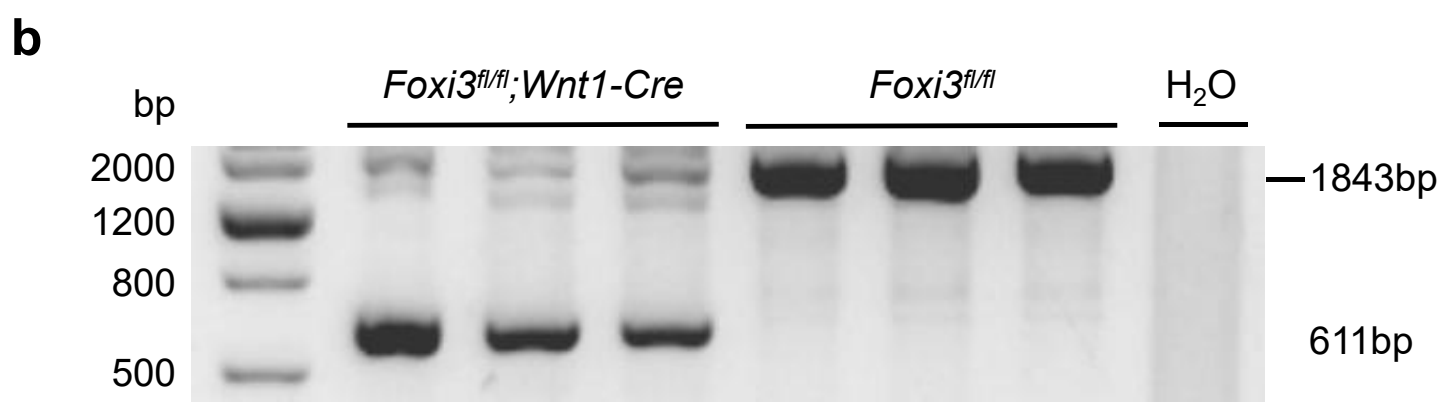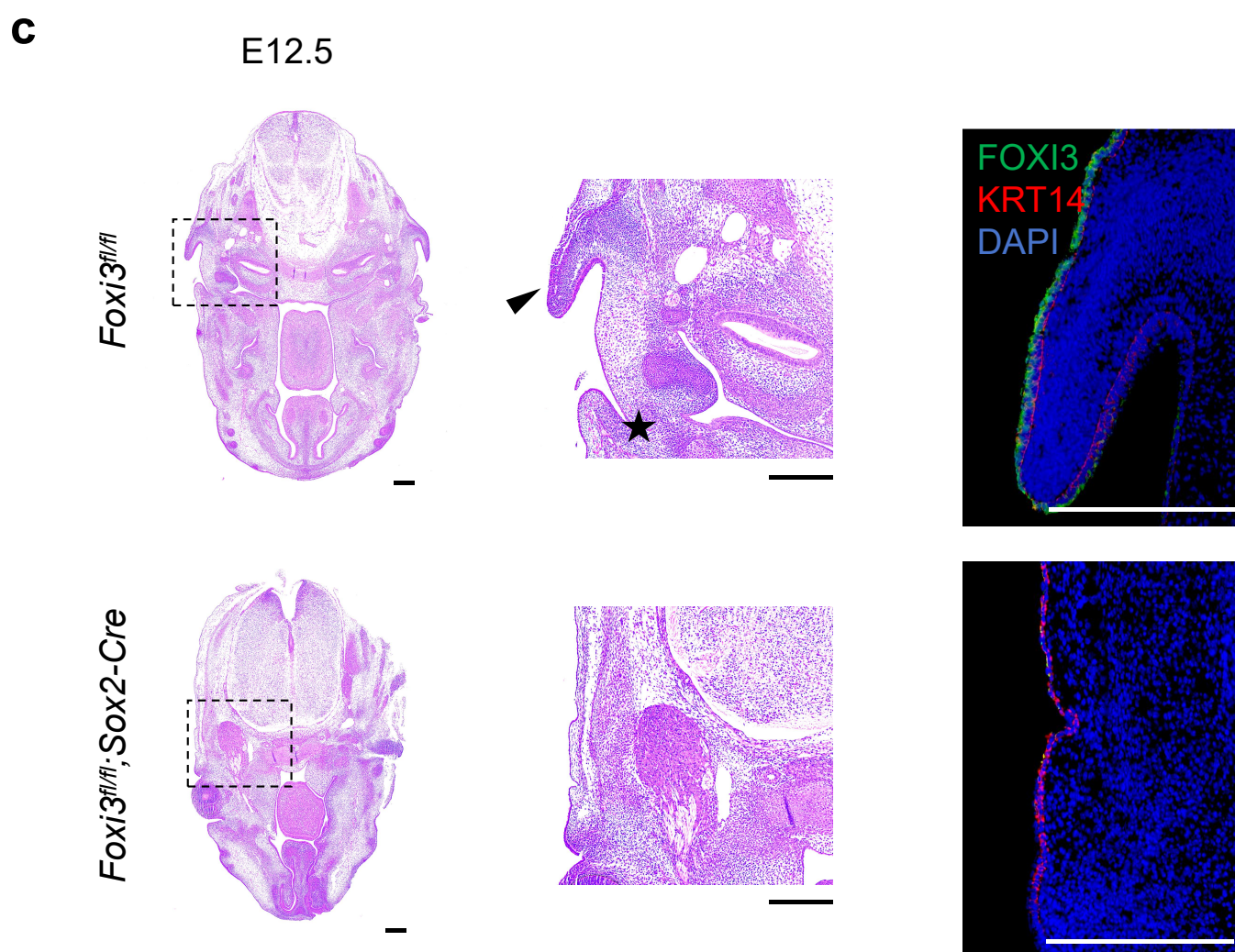

Supplement: Supplementary file 2 — Supplemental Figure 1 [file 41413_2025_499_MOESM2_ESM.pdf]

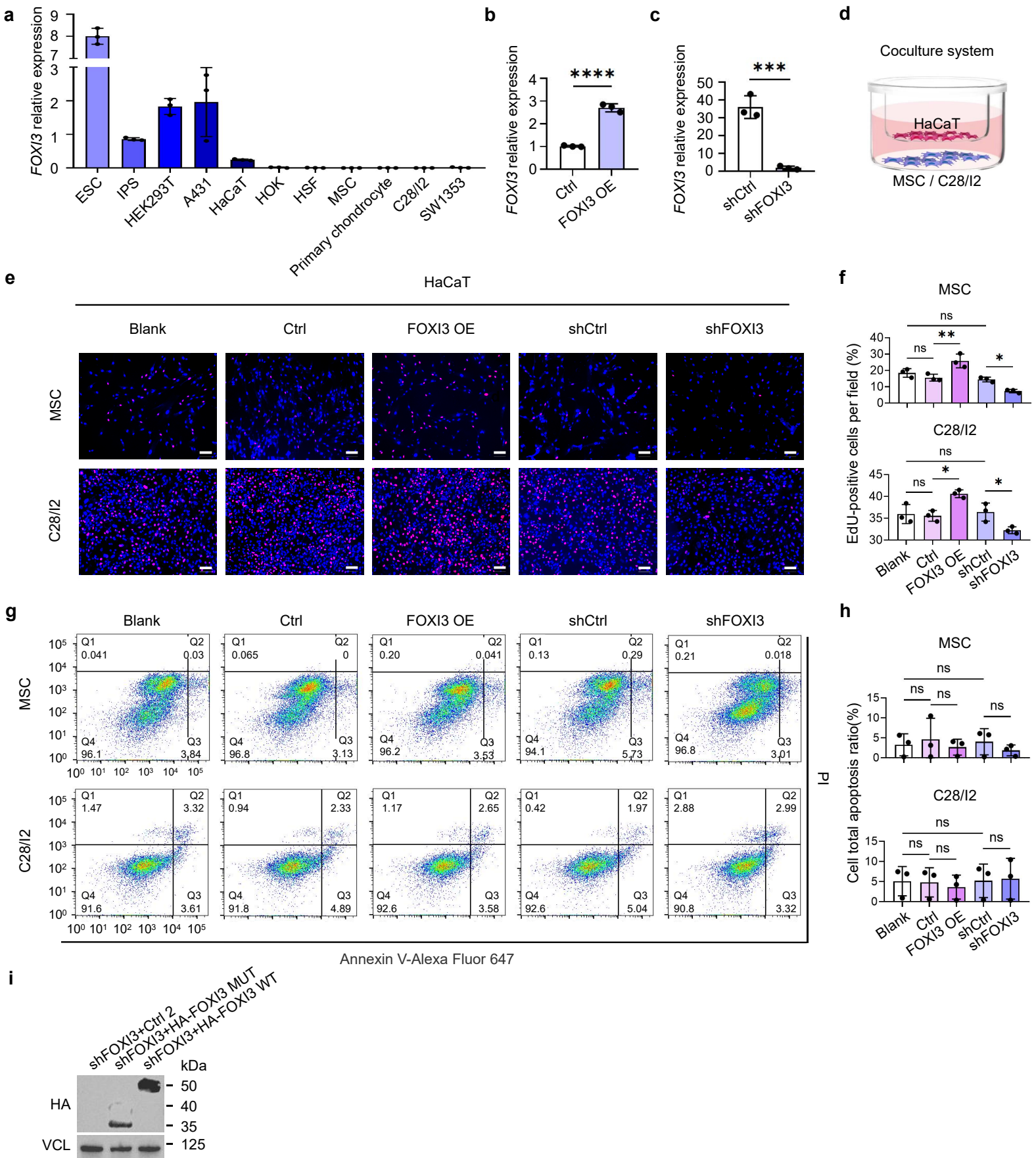

Supplement: Supplementary file 3 — Supplemental Figure 2 [file 41413_2025_499_MOESM3_ESM.pdf]

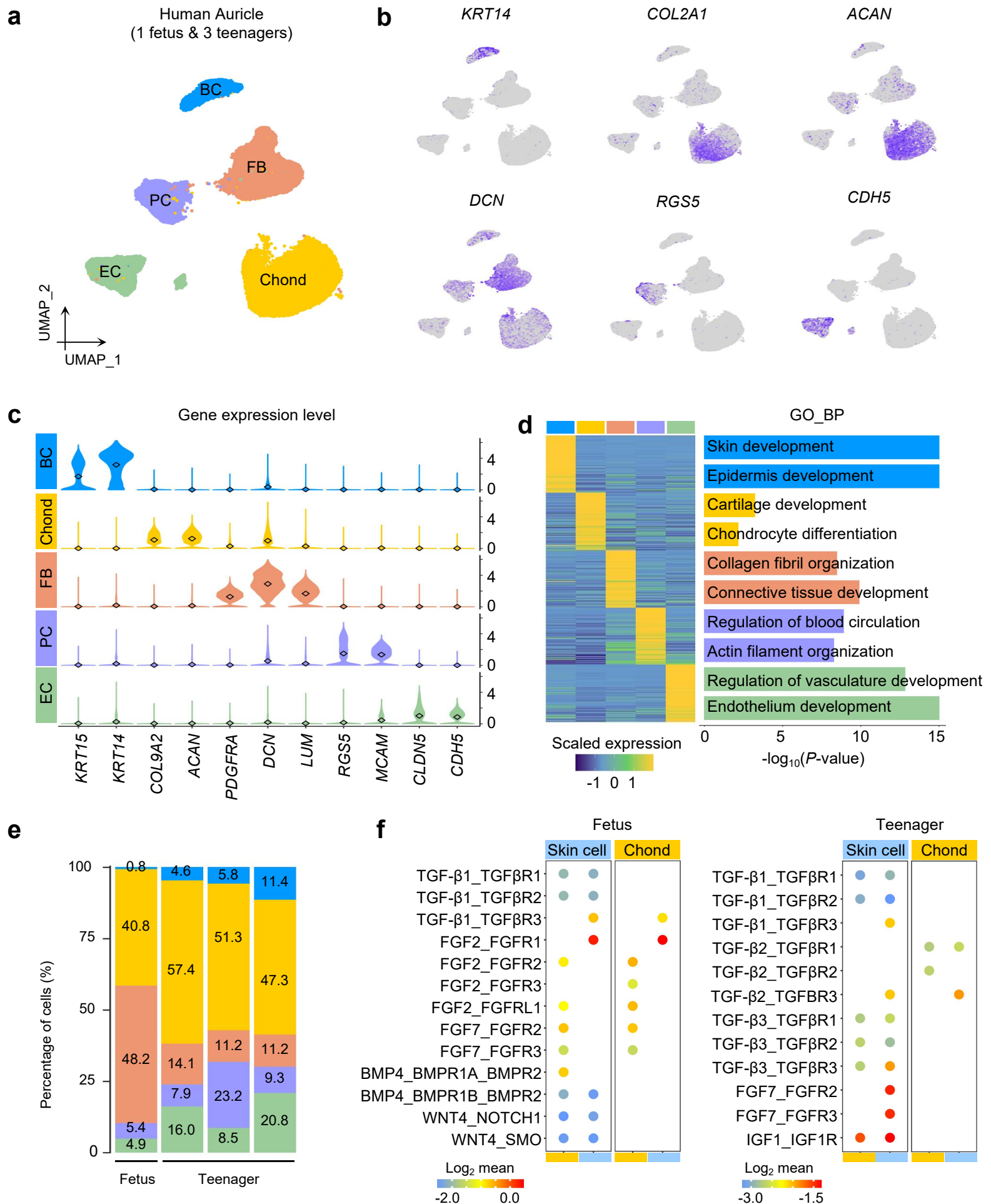

Supplement: Supplementary file 4 — Supplemental Figure 3 [file 41413_2025_499_MOESM4_ESM.pdf]

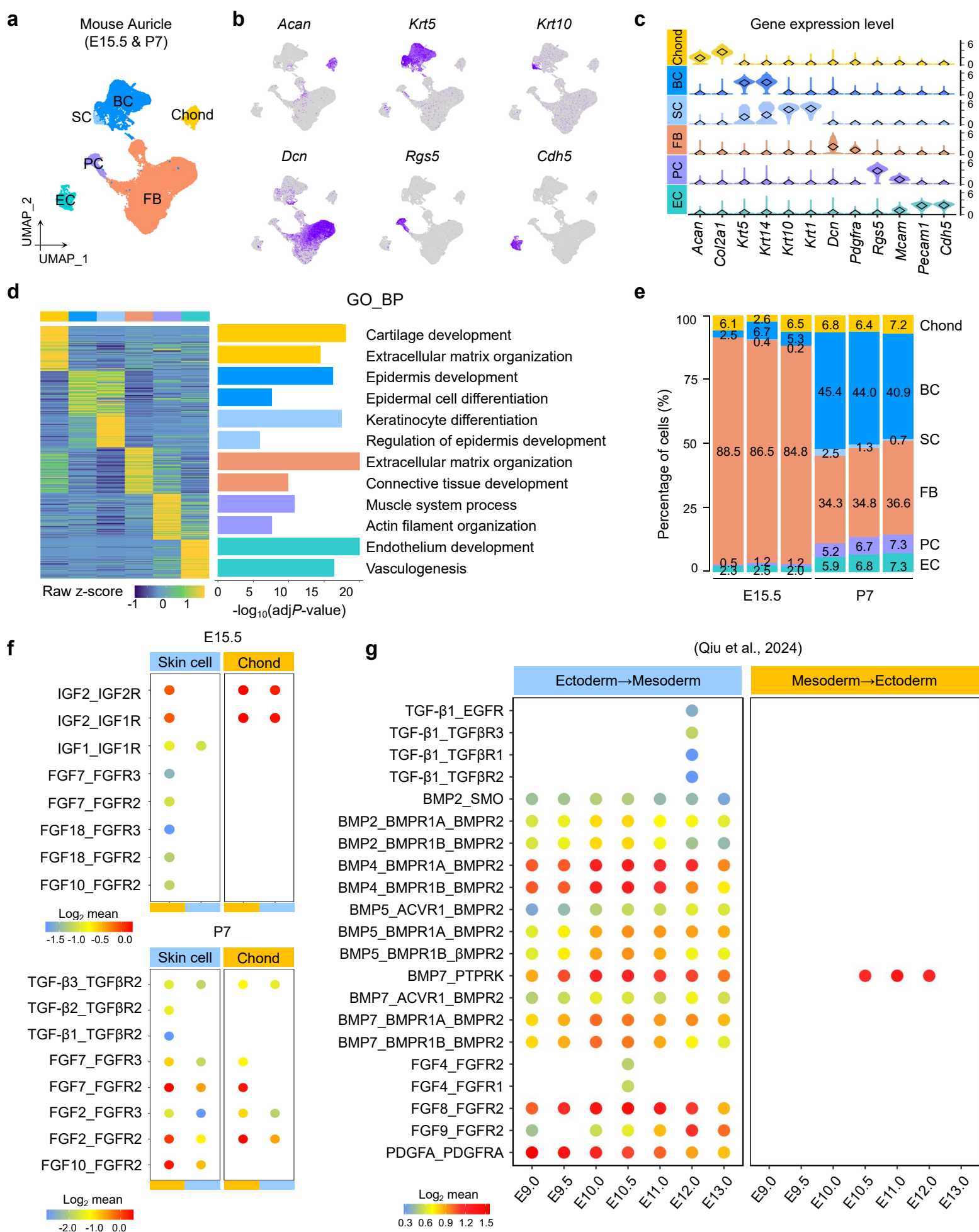

Supplement: Supplementary file 5 — Supplemental Figure 4 [file 41413_2025_499_MOESM5_ESM.pdf]

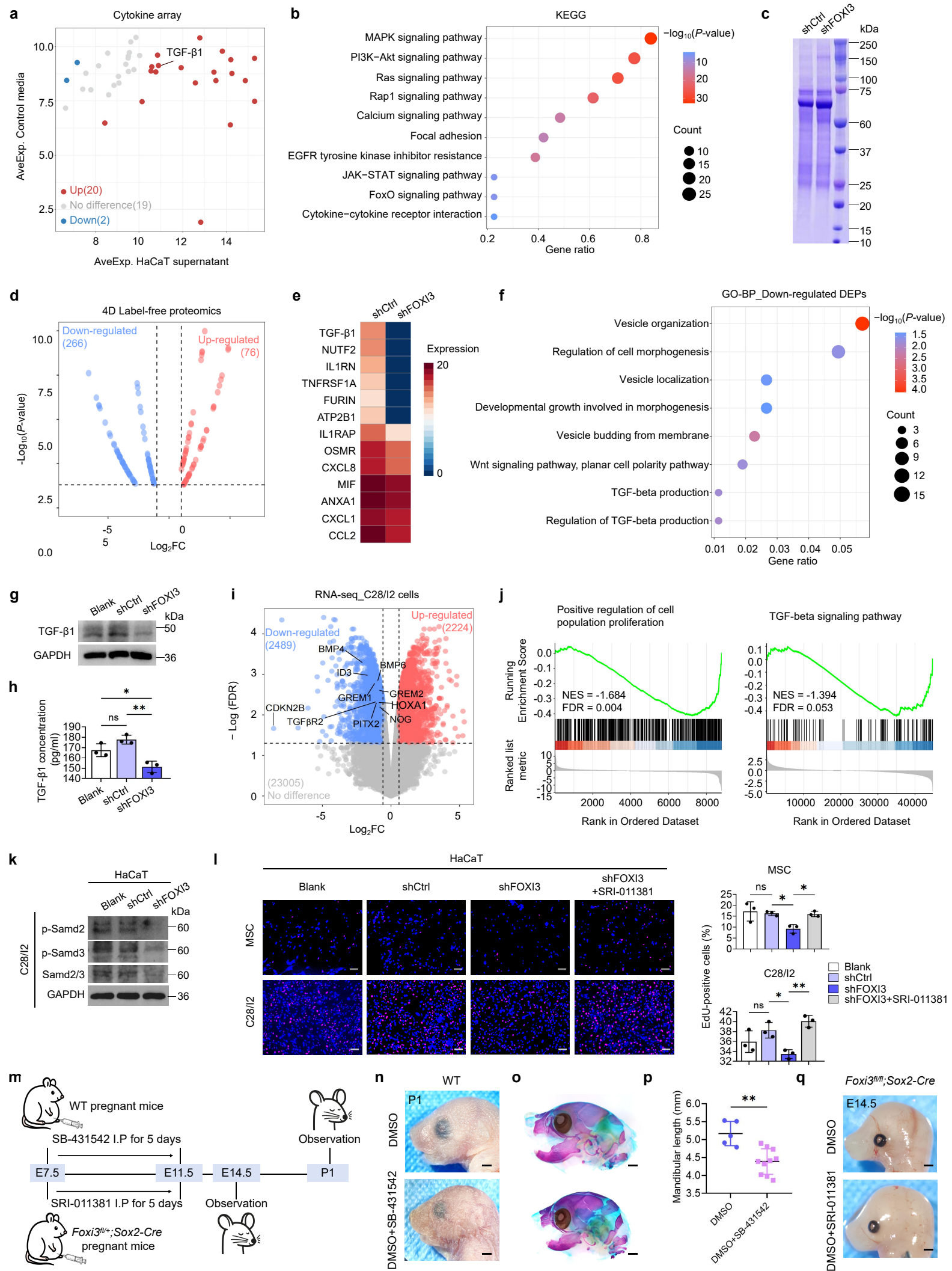

Supplement: Supplementary file 6 — Supplemental Figure 5 [file 41413_2025_499_MOESM6_ESM.pdf]

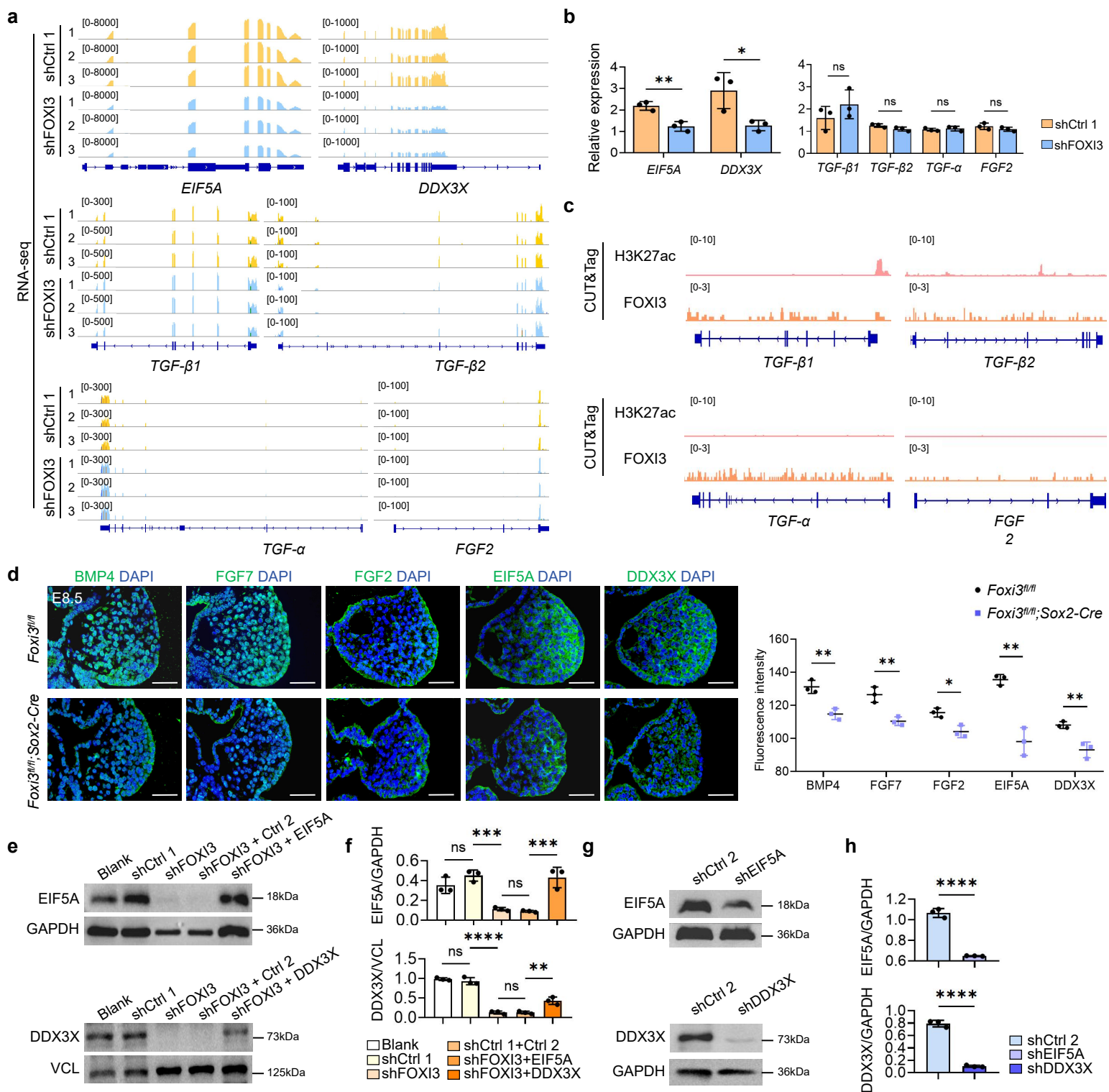

Supplement: Supplementary file 7 — Supplemental Figure 6 [file 41413_2025_499_MOESM7_ESM.pdf]

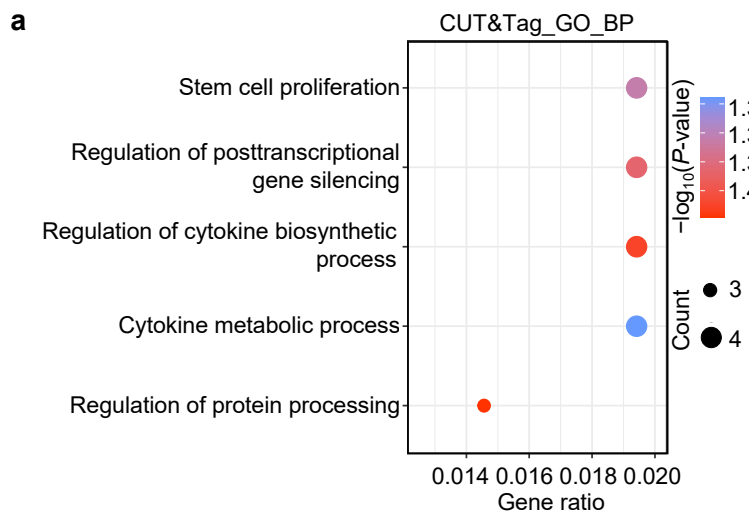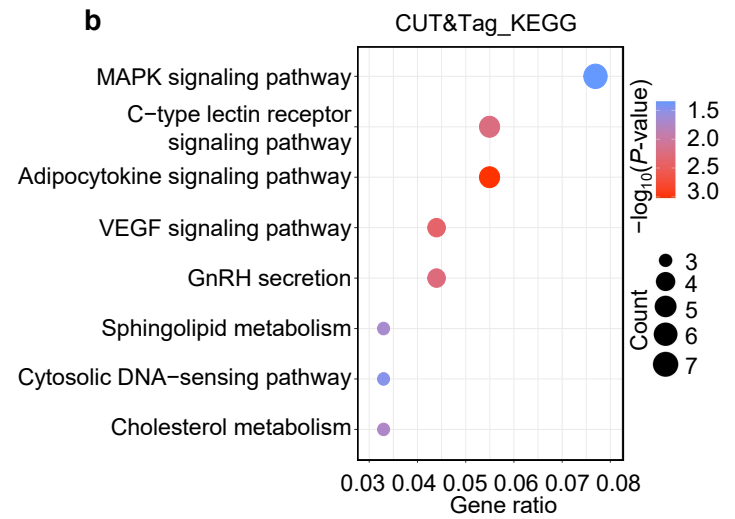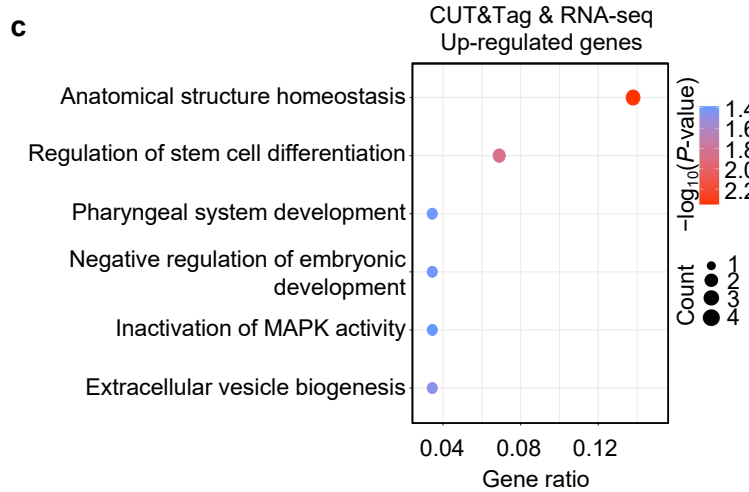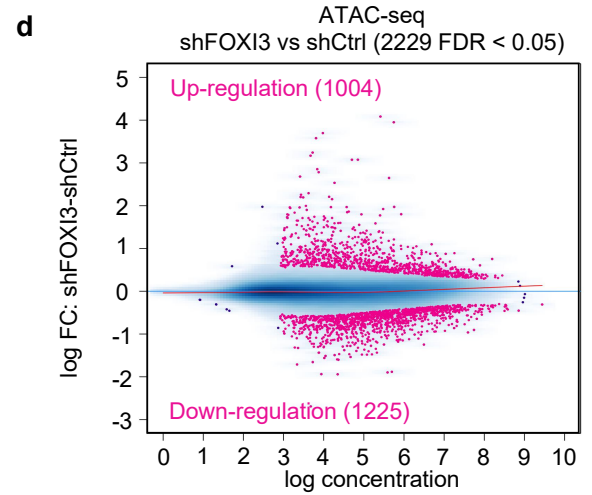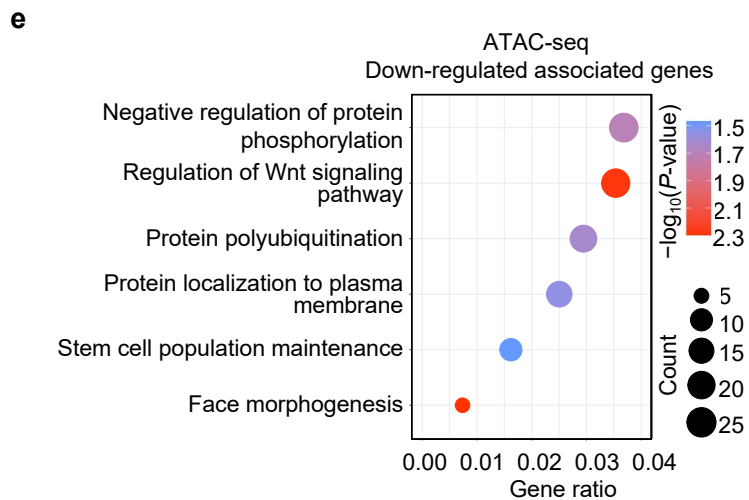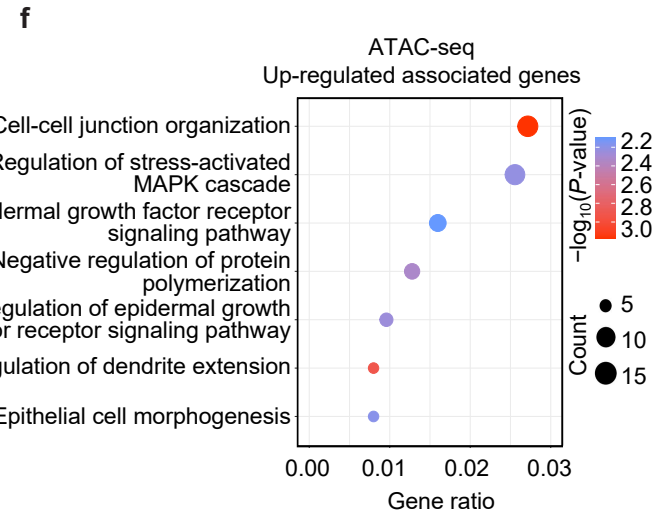

Supplement: Supplementary file 8 — Supplemental Figure 7 [file 41413_2025_499_MOESM8_ESM.pdf]

c.941delC

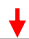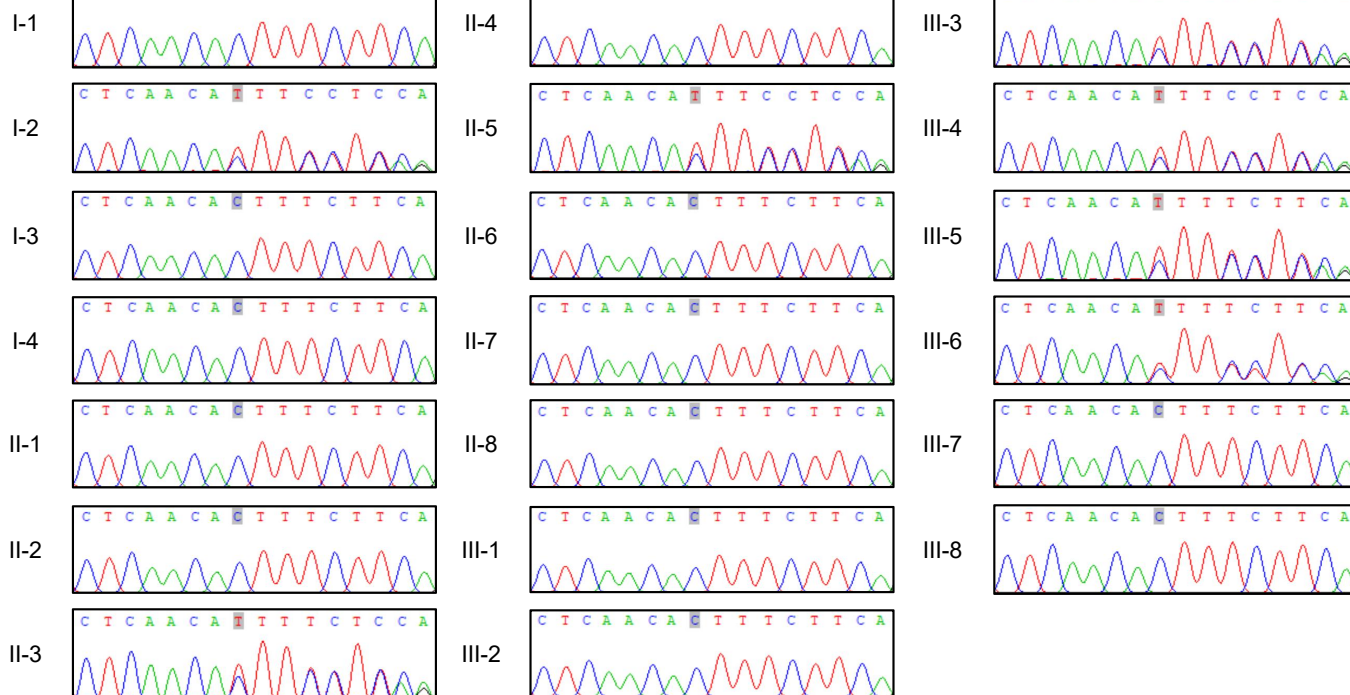

Supplement: Supplementary file 9 — Supplemental Figure 8 [file 41413_2025_499_MOESM9_ESM.pdf]

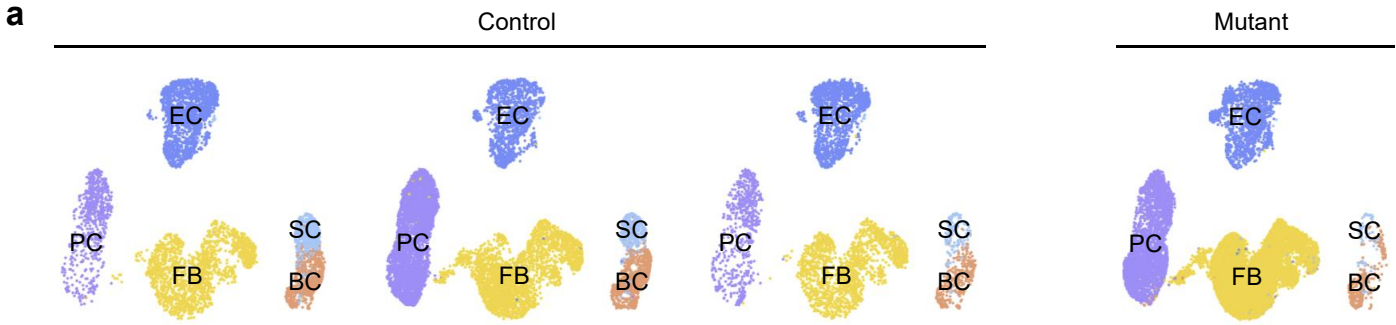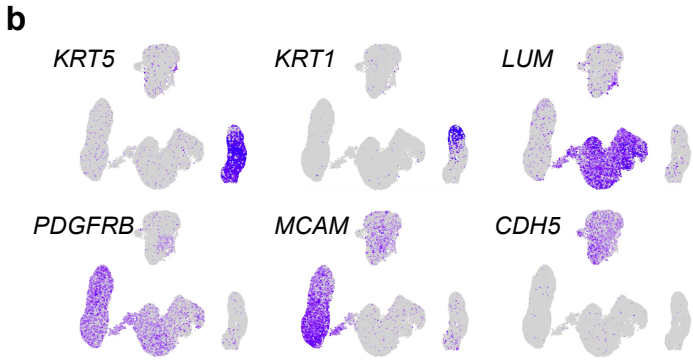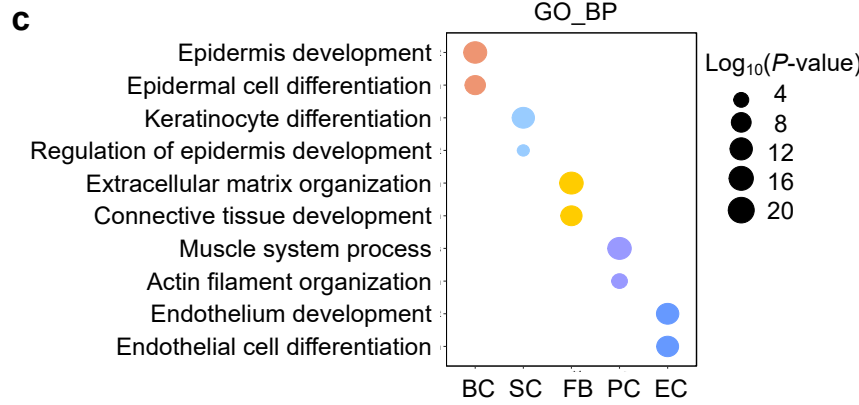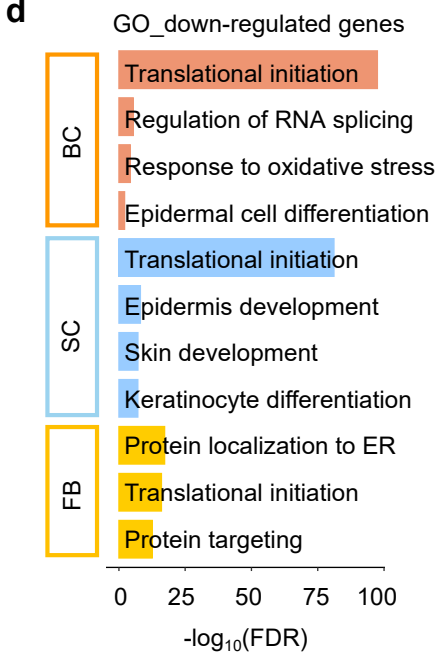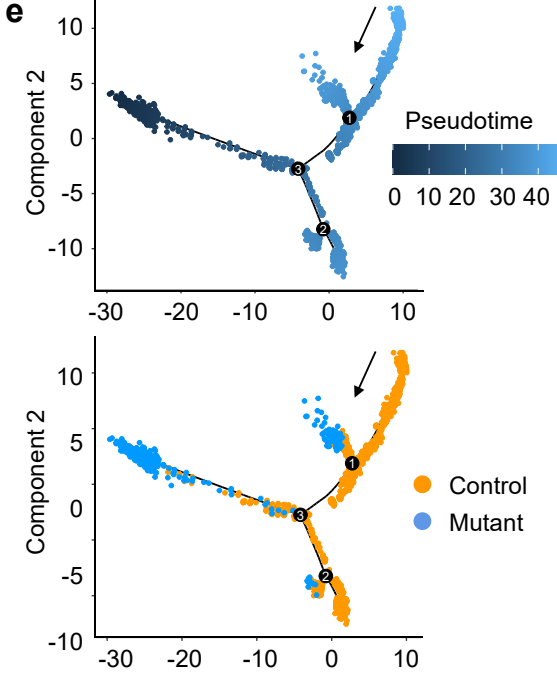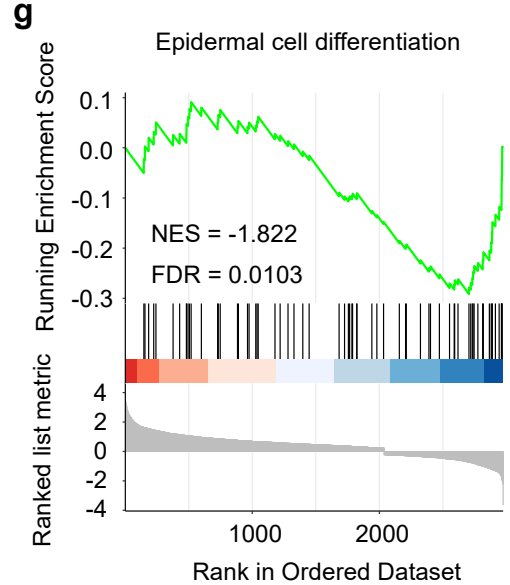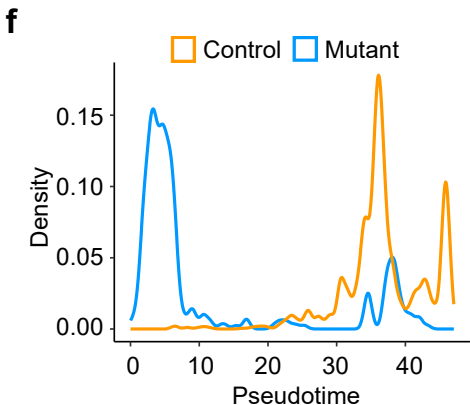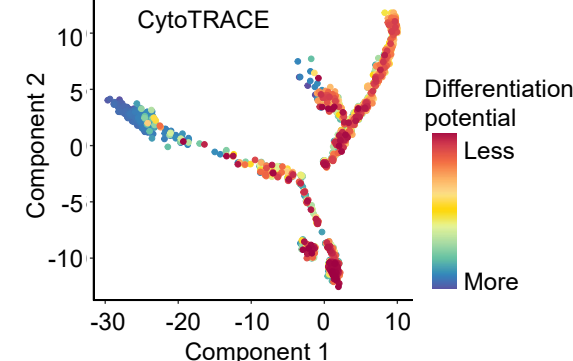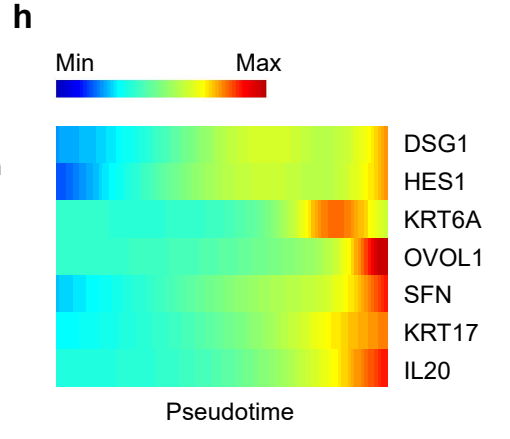

Supplement: Supplementary file 10 — Supplemental Figure 9 [file 41413_2025_499_MOESM10_ESM.pdf]

**a**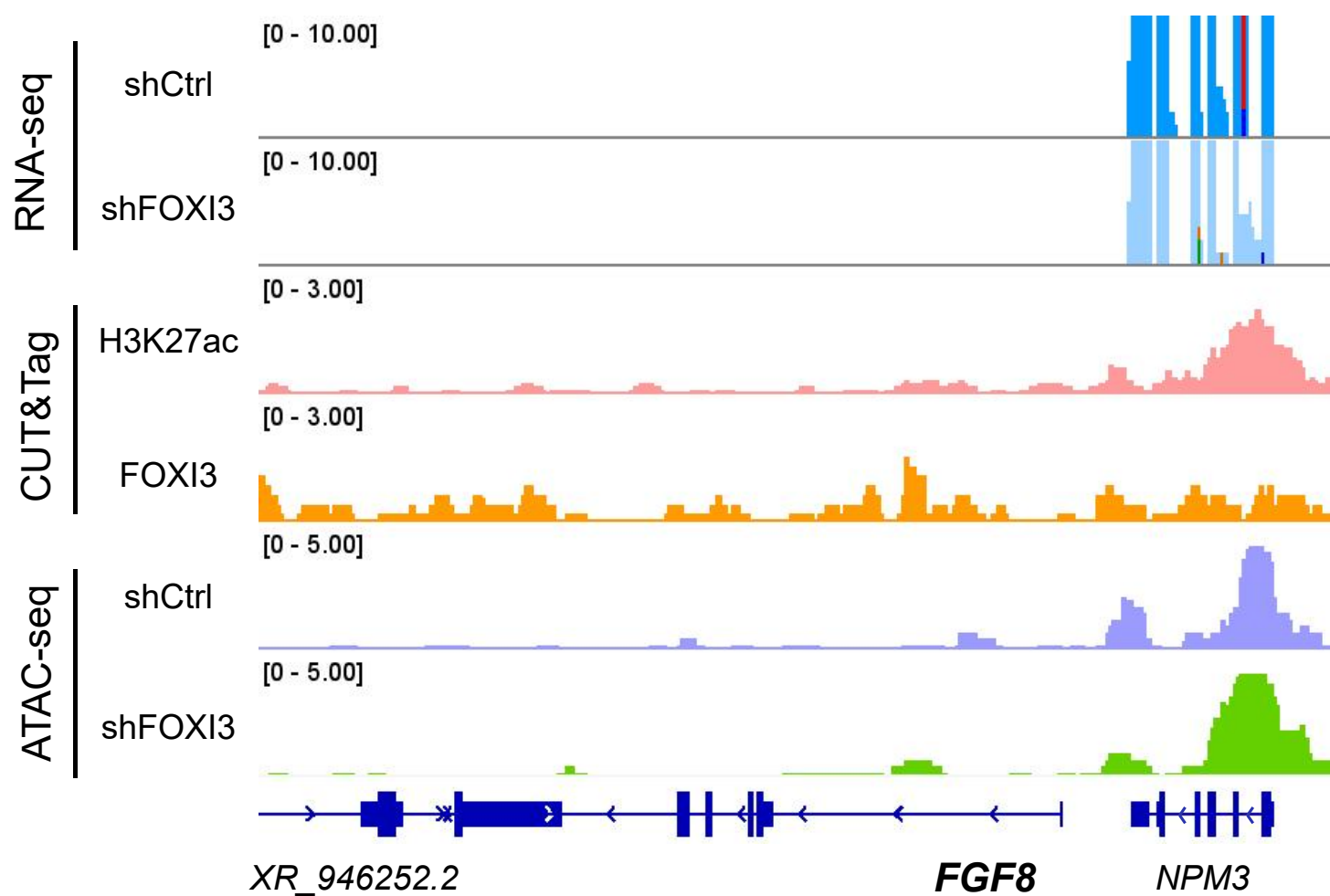**b**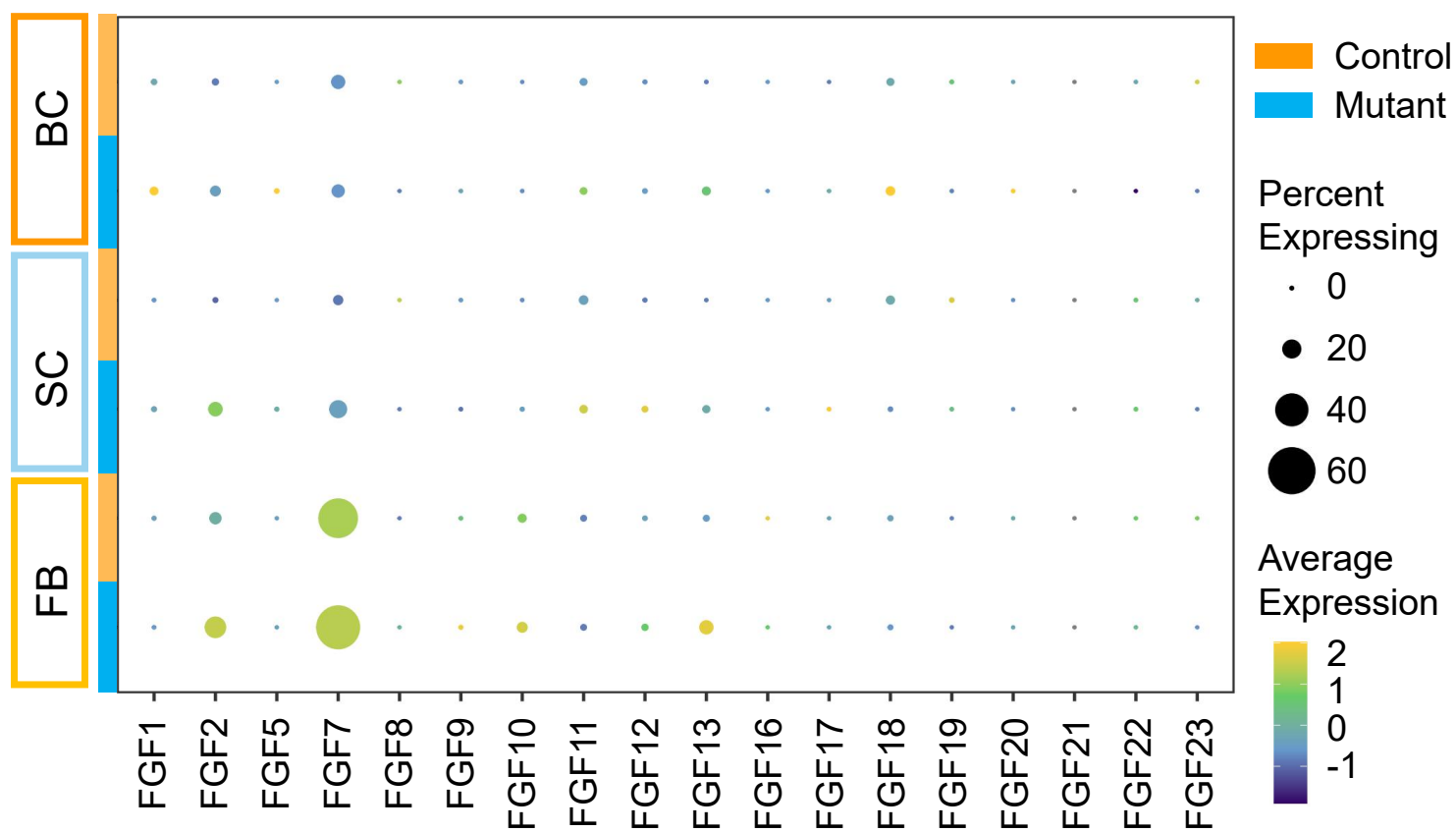

Supplement: Supplementary file 11 — Supplemental Figure 10 [file 41413_2025_499_MOESM11_ESM.pdf]
