# Supplementary material for: FOXI3 establishes the ectodermal niche in pharyngeal arches for cranial neural crest cells and their lineages
Source: Bone Res. 2026 Feb 4;14:16. doi: 10.1038/s41413-025-00499-w (PMC12873258; doi:10.1038/s41413-025-00499-w)
Supplement: Supplementary file 13 — Supplemental Figure Legends [file 41413_2025_499_MOESM13_ESM.docx]

**Fig. S1** Detection of Cre enzyme activity in *Foxi3* conditional knockout mice. **a** Schematic diagram of the Cre-loxP system. **b** Gel electrophoresis showing Cre enzyme activity in the auricle cartilage of *Foxi3*^fl/fl^; *Wnt1-Cre* mice and control littermates. DEPC water was used as a negative control. Some bands without Cre activity were detected, potentially due to the presence of cartilage membranes during tissue retrieval. **c** H&E staining of *Foxi3*^fl/fl^*; Sox2-Cre* mice shows the absence of external ear structures at E12.5, compared to controls. Immunofluorescence staining reveals FOXI3 knockout in the auricle epidermis (marked with KRT14) of *Foxi3*^fl/fl^; *Sox2-Cre* mice at E12.5. Arrows indicate auricle; star indicates external auditory canal. Scale bars: 50 µm.

**Fig. S2** FOXI3 expression in cell lines and its effects on chondrocyte proliferation but not apoptosis in co-culture. **a** qPCR detection of *FOXI3* expression in different cell lines. *FOXI3* is not expressed in mesenchymal stem cells (MSC) or chondrocytes (primary chondrocyte, C28/I2, SW1353), but is highly expressed in early embryonic cells (human embryonic stem cells (ESC), induced pluripotent stem (IPS) cells, human embryonic kidney 293 cells (HEK293T)) and epidermal cells (A431, HaCaT). There are three biological replicates per cell line. **b-c** qPCR assays showing the efficiency of FOXI3 overexpression (OE) and knockdown (shFOXI3) in HaCaT cells. **d** Diagram of the transwell coculture system used for assessing MSC and C28/I2 cells’ proliferation and apoptosis when co-cultured with HaCaT cells. **e** Proliferation of MSCs and C28/I2 cells after three days of co-culture with HaCaT cells, assessed using EdU assay (red). **f** Quantification of EdU assay results. **g** Apoptosis analysis via flow cytometry using Fluor 647 conjugated Annexin V and PI double-staining in MSCs and C28/I2 cells after co-culture with HaCaT cells. **h** Statistical analysis of apoptotic MSCs and C28/I2 cells. **i** Efficiency of overexpressing HA-FOXI3 wild-type (WT) or mutant (MUT) in *FOXI3*-knockdown HaCaT cells. Statistical significance was analyzed via unpaired two-tailed Student’s *t* test for (**b, c**), one-way ANOVA with Tukey’s multiple comparison test for (**f, h**). ns, no significance; ^*^*p* < 0.05, ^**^*p* < 0.01, ^***^*p* < 0.001, and ^****^*p* < 0.0001

**Fig. S3** Strong cell-to-cell communication between auricle skin cells and chondrocytes in human auricle development. **a** UMAP of single cells from auricle from one human fetus at 13 weeks gestation and three teenagers. Distinct cell populations are represented in different colors. EC, endothelial cell; Chond, chondrocyte; BC, basal cell; SC, spinous cell; FB, fibroblast; PC, pericyte. **b** Feature plots demonstrating the expression distribution for selected cluster-specific genes. Each cell’s expression levels are color-coded and overlaid onto the UMAP plot, with color depth indicating expression intensity. **c** Violin plots displaying normalized expression levels of selected marker genes for each cell type. The median value is represented by a diamond within each violin. **d** Heatmap (left panel) and GO-BP enrichment analysis (right panel) of DEGs in each cell cluster. **e** Stacked bar charts illustrating the relative proportions of six subtypes in each auricle sample. **f** Dot plot showing selected ligand-receptor interactions in human auricle chondrocytes and skin cells. Color intensity indicates the expression levels of ligand-receptor pairs, with blue representing relatively weak expression and orange indicating stronger expression.

**Fig. S4** Cytokine-mediated communication between ectoderm-derived skin cells and chondrocytes differentiated from the mesoderm during mouse auricle development. **a** UMAP visualization of single cells from E15.5 and P7 mouse auricles, with distinct cell populations labeled with different colors. Three biological replicates are represented in each group. Chond, chondrocyte; BC, basal cell; SC, spinous cell; FB, fibroblast; PC, pericyte; EC, endothelial cell. **b** Feature plots illustrating the expression distribution of selected cluster-specific genes. Each cell’s expression levels are color-coded and overlaid onto the UMAP plot with color depth reflecting expression intensity. **c** Violin plots displaying normalized expression levels of selected marker genes for each cell type, with the median value indicated by a diamond within each violin. **d** Heatmap (left panel) and GO-BP enrichment analysis (right panel) of DEGs in each cell cluster. **e** Stacked bar charts showing the relative proportions of six subtypes in E15.5 and P7 mice auricles. **f** Dot plot depicting selected chondrogenesis-related ligand-receptor interactions in E15.5 and P7 mouse auricle chondrocytes and skin cells, with dot color indicating interaction score. **g** Dot plot illustrating strong cellular communication related to chondrogenesis between ectodermal cells and neural crest-derived mesodermal cells during earlier developmental stages (E9.0 to E13), based on whole mouse embryo scRNA-seq data.

**Fig. S5** FOXI3 in epidermal cells affects chondrogenesis-related cytokine levels to regulate chondrocyte proliferation. **a** Cytokine array in control medium and HaCaT cell supernatants. One sample per group for one-on-one comparison. FC > 1.5. **b** KEGG analysis of differentially expressed cytokines (*p* < 0.05). **c** SDS-PAGE of secreted proteins from FOXI3-knockdown HaCaT cell supernatants. **d-f** 4D label-free proteomics identifying altered secreted proteins: (**d**) volcano plot (FC > 2, p < 0.05); (**e**) cytokine heatmap (dark blue, undetected); (**f**) GO enrichment of downregulated DEPs (p < 0.05). **g-h** WB (**g**) and ELISA (**h**) detection of intracellular and extracellular TGF-β1 in FOXI3-knockdown HaCaT cells. **i-j** Transcriptomic analysis of C28/I2 cells co-cultured with FOXI3-knockdown HaCaT cells: (**i**) volcano plot of DEGs (FC > 1.5, FDR < 0.05); (**j**) GSEA showing downregulated GO-BP terms. **k** WB showing reduced phosphorylation of SMAD2 and SMAD3 in C28/I2 cells co-cultured with FOXI3-knockdown HaCaT cells compared with control co-cultures. **l** EdU assay and quantification showing proliferation of MSCs and C28/I2 cells co-cultured with HaCaT cells. Scale bars: 100 µm. **m** Schematic of intraperitoneal injection (I.P) and observation timeline. Wild-type (WT) pregnant mice received SB-431542 (E7.5-E11.5; analyzed at P1). After mating with *Foxi3*^fl/+^;*Sox2-Cre* males, pregnant mice received SRI-011381 (E7.5-E11.5; analyzed at E14.5). **n-o** Bright-field (**n**) and Alizarin Red-Alcian Blue staining (**o**) of WT mice treated with DMSO or SB-431542. Scale bars indicate equivalent magnification across samples. **p** Mandibular length quantification of P1 WT mice showing reduced length after SB-431542 treatment. **q** Bright-field images of *Foxi3*^fl/fl^;*Sox2-Cre* mice show partial improvement of craniofacial skeletal malformations after SRI-011381 treatment. Scale bars indicate equal magnification. Statistical analysis: one-way ANOVA with Tukey’s post hoc test for (**h, l**); unpaired two-tailed Student’s *t* test for (**p**). ns, no significance; ^*^*p* < 0.05, ^**^*p* < 0.01

**Fig. S6** Changes in target genes and cytokines in FOXI3-knockdown HaCaT cells and *Foxi3*^fl/fl^; *Sox2-Cre* mice. **a** Visualization of target genes (*EIF5A*, *DDX3X*) and cytokine-encoding genes (*TGF-β1*, *TGF-β2*, *TGF-α*, *FGF2*) in RNA-seq data using IGV software. Three biological replicates were performed for each group. **b** qPCR confirmation of *EIF5A*, *DDX3X*, *TGF-β1*, *TGF-β2*, *TGF-α*, and *FGF2* expression in FOXI3 knocked-down HaCaT cells. Three biological replicates were performed for each group. **c** Normalized CUT&Tag profiles at cytokine-encoding gene loci (*TGF-β1*, *TGF-β2*, *TGF-α*, *FGF2*) showing that FOXI3 does not bind to their promoters. **d** Immunofluorescence staining and quantitative fluorescence analysis of E8.5 mouse sections demonstrating significantly decreased expression of several cytokines and the two target genes following *Foxi3* knockout in the auricle ectoderm. Scale bars: 100 µm. **e-f** Western blot assays (**e**) and grayscale statistics (**f**) demonstrating the efficiency of EIF5A and DDX3X overexpression in FOXI3-knockdown HaCaT cells. **g-h** WB assays (**g**) and grayscale statistics (**h**) showing the efficiency of EIF5A and DDX3X knockdown in HaCaT cells. Statistical significance was analyzed via unpaired two-tailed Student’s *t* test for (**b, d, h**), one-way ANOVA with Tukey’s multiple comparison test for (**f**). ns, no significance; ^*^*p* < 0.05, ^**^*p* < 0.01, ^***^*p* < 0.001, and ^****^*p* < 0.0001

**Fig. S7** Analysis of CUT&Tag, RNA-seq, and ATAC-seq. **a** GO analysis of genes with promoter regions bound by FOXI3. **b** KEGG enrichment analysis of genes with FOXI3-bound promoter regions. **c** GO analysis of upregulated target genes in FOXI3-knockdown HaCaT cells. **d** Volcano plot showing differentially accessible peaks between control and FOXI3-knockdown HaCaT cells. **e** GO analysis of genes associated with downregulated chromatin accessibility in FOXI3-knockdown HaCaT cells. **f** GO analysis of genes with upregulated chromatin accessibility in FOXI3-knockdown HaCaT cells.

**Fig. S8** Sanger sequencing of all collected individuals in the CFM pedigree. Red arrow points to the *FOXI3* mutation site, highlighted in gray.

**Fig. S9** Additional single-cell RNA sequencing analysis results from human control and mutant auricle skin samples. **a** UMAP visualization of single cells from mutant and three control auricle skin samples. Different colors label distinct cell populations. BC, basal cell; SC, spinous cell; FB, fibroblast; PC, pericyte; EC, endothelial cell. **b** Feature plots showing the distribution of expression for selected cluster-specific genes. Expression levels for each cell are color-coded and overlaid onto the UMAP plot. The color depth indicates the expression level. **c** GO enrichment analysis of DEGs for each cell cluster. **d** GO enrichment analysis of downregulated DEGs in BCs, SCs, and FBs for both control and mutant samples. **e** Plots showing the pseudotime trajectories of BCs in the control and mutant samples; the first column shows clusters colored by pseudotime, the second column shows clusters colored by the control and mutant groups, and the final column marks the differentiation potential of these BCs. **f** Density distribution of BCs along the pseudotime trajectory in control and mutant samples. **g** GSEA showing downregulated genes enriched in the BP of epidermal cell differentiation in mutant BCs. **h** Heatmap showing the upregulated DEGs related to epidermal cell differentiation in BCs with the color key ranging from blue (low expression) to red (high expression).

**Fig. S10** *FOXI3* frameshift mutation does not directly affect FGF signaling in the auricle skin. **a** Normalized RNA-seq, CUT&Tag, and ATAC-seq profiles at the *FGF8* locus. FOXI3 does not bind directly to *FGF8*, and the downregulation of FOXI3 does not alter *FGF8* mRNA expression. **b** Dot plot illustrating no significant differences in the expression of FGF signals among different skin cell types between the patient and control. The dot size represents the fraction of cells expressing a particular marker, and the color intensity corresponds to mean normalized scaled expression levels. BC, basal cell; SC, spinous cell; FB, fibroblast.

**Fig. S11** *FOXI3* transcripts were not captured by bulk or single-cell RNA-seq because of high GC content. **a** Visualization of *FOXI3* expression in RNA-seq using IGV software, with three replicates per group. **b** Color-coded expression levels of *FOXI3* for each cell, overlaid on the UMAP plot. The depth of color represents the expression level. Chond, chondrocyte; BC, basal cell; SC, spinous cell; FB, fibroblast; PC, pericyte; EC, endothelial cell. **c** The mRNA sequence of human *FOXI3*, with G and C bases highlighted in blue. GC-content: 67%.
