# Supplementary material for: FOXI3 establishes the ectodermal niche in pharyngeal arches for cranial neural crest cells and their lineages
Source: Bone Res. 2026 Feb 4;14:16. doi: 10.1038/s41413-025-00499-w (PMC12873258; doi:10.1038/s41413-025-00499-w)
Supplement: Supplementary file 12 — Supplemental Figure 11 [file 41413_2025_499_MOESM12_ESM.pdf]

a

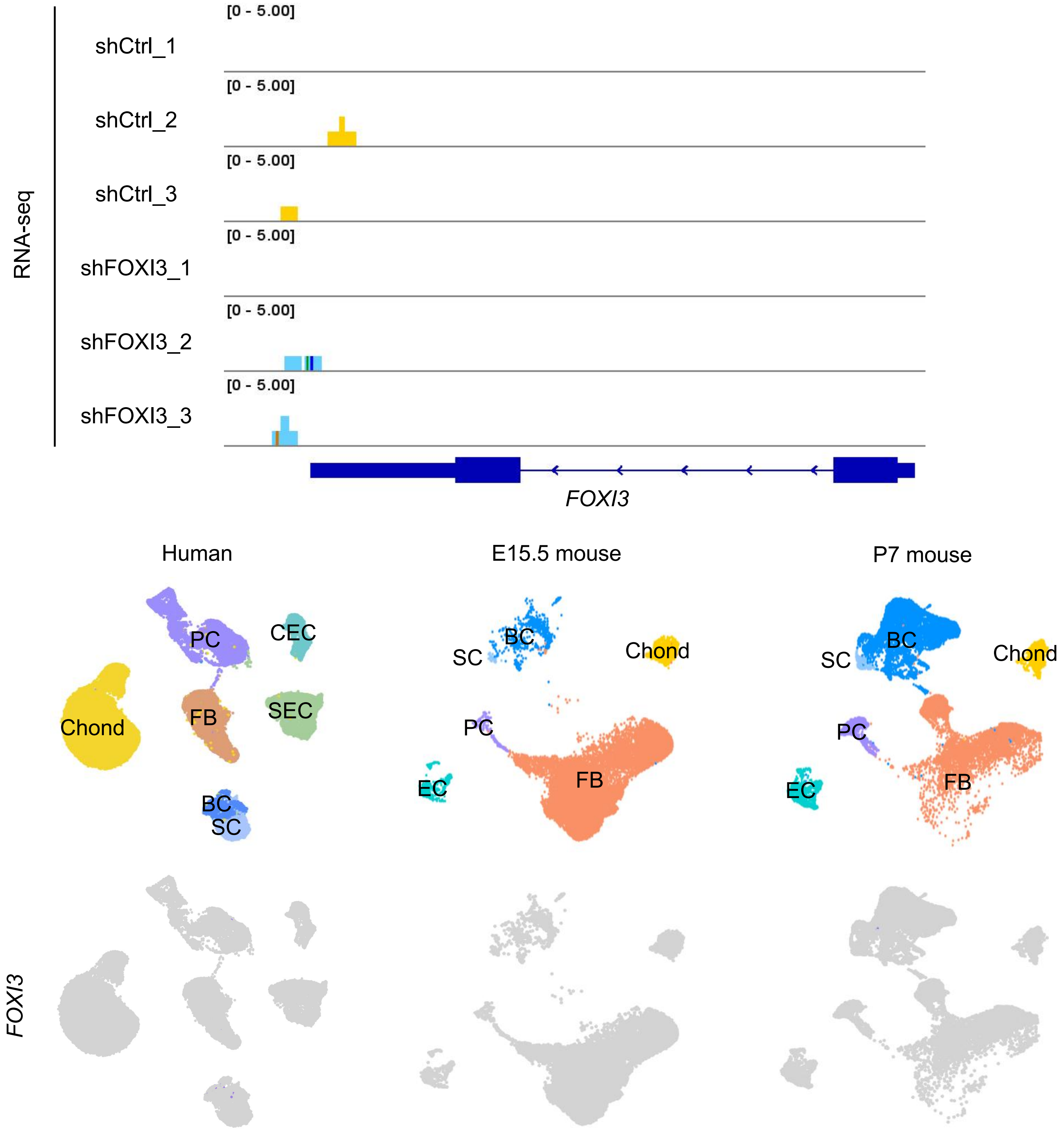

c

>NM_001135649.3:159-1421 Homo sapiens forkhead box I3 (FOXI3), mRNA
ATGGCCCTCTACTGCGGCGACAACTTCGGAGTGTATTCGCAGCCCGGCCTGCCCCCGCCCGCCGCCACCGCCGC
CGCCCCGGGCGCCCCCCCGGCAGCCAGGGCGCCTTACGGGCTGGCCGACTACGCCGCGCCGCCGGCCGCCGC
CGCCAACCCCTACCTGTGGCTCAACGGGCCCGGCGTGGGAGGCCCGCCCTCCGCCGCCGCCGCAGCCGCCGCC
GCGTACCTGGGCGCTCCGCCGCCGCCGCCGCCCCCGGGGCCGCGGCCGGGCCCTTTCTGCAGCCACCGCCTG
CCGCCGGCACCTTCGGCTGCTCTCAGCGGCCCTTCGCGCAGCCCGCGCCCGCCGCGCCCGCCTCGCCCGCCGC
GCCCGCGGGGCCCGGGGAGCTGGGCTGGCTGTCCATGGCCAGCCGCGAGGACCTGATGAAGATGGTGCGGCCG
CCCTACTCGTATTCGGCGCTCATCGCCATGGCCATTCAGAGCGCGCCCGAGCGCAAACTCACTCTCAGCCACATCT
ACCAGTTCGTCGCCGATAGCTTCCCCTTCTACCAGCGCAGCAAGGCCGGCTGGCAGAACTCCATCCGCCACAACCT
GTCGCTCAACGACTGCTTCAAGAAGGTGCCCCGCGACGAGGACGACCCAGGAAAGGGTAATTATTGGACTCTTGAT
CCGAACTGCGAGAAAATGTTTGACAATGGGAACTTCCGTCGGAAGCGAAAGCGCCGCTCTGAGGCCAGCAATGGC
TCCACAGTGGCTGCTGGGACATCAAAGTCCGAAGAAGGGCTCTCCTCAGGATTGGGGTCTGGAGTGGGTGGGAAG
CCAGAAGAAGAGAGCCCCTCCACTCTGCTGAGGCCTTCCCACTCCCCAGAGCCTCCGGAGGGCACCAAGAGTACT
GCCTCATCTCCTGGAGGACCCATGCTCACCTCCACCCCTTGTCTCAACACTTTCTTCAGCAGCCTCAGCTCCTTGAG
TGTCAGCAGCAGTGTGAGTACCCAGCGGGCTCTCCCTGGCAGCCGCCACCTAGGGATCCAGGGGGCCCAGCTGC
CCTCCAGCGGCGTGTTCTCCCCGACCTCCATCTCAGAGGCCTCAGCAGACACCTTGCAACTGAGCAATAGCACCAG
CAATAGCACCGGCCAGAGATCTTCCTATTACAGCCCTTTCCCTGCCAGCACCAGCGGGGGGCAAAGCAGCCCCTTC
AGCAGCCCTTTCCACAACTTTAGCATGGTGAACAGCCTCATCTACCCCCGAGAGGGCTCCGAGGTGTAG
